# Sirtuin 3 reinforces acylcarnitine metabolism and maintains thermogenesis in brown adipose tissue of aging mice

**DOI:** 10.1111/acel.14332

**Published:** 2024-09-30

**Authors:** Kuiliang Zhang, Yucheng Wang, Yujie Sun, Lamei Xue, Yu Wang, Chenzhipeng Nie, Mingcong Fan, Haifeng Qian, Hao Ying, Li Wang, Yan Li

**Affiliations:** ^1^ State Key Laboratory of Food Science and Technology, School of Food Science and Technology Jiangnan University Wuxi China; ^2^ Xuhui Central Hospital of Shanghai Shanghai China; ^3^ CAS Key Laboratory of Nutrition, Metabolism and Food Safety, Shanghai Institute of Nutrition and Health, Chinese Academy of Sciences University of Chinese Academy of Sciences Shanghai China

**Keywords:** acylcarnitine metabolism, aging, brown adipose tissue, Sirt3, thermogenesis

## Abstract

Acylcarnitine (ACar) is a novel fuel source for activating thermogenesis in brown adipose tissue (BAT). However, whether ACar metabolism underlies BAT thermogenesis decline with aging remain unclear. Here, the L‐carnitine‐treated young and aging mice were used to investigate the effects of activation of ACar metabolism on BAT thermogenesis during aging. We showed that long term L‐carnitine feeding, which results in an elevation in circulating ACar levels, failed to improve cold sensitivity of aging mice, which still displayed impaired thermogenesis and ACar metabolism in interscapular BAT (iBAT). The RNA‐sequencing was used to identify the key regulator for the response of aging mice to LCar induced activation of ACar metabolism in BAT, and we identified Sirt3 as a key regulator for the response of aging mice to L‐carnitine induced activation of ACar metabolism in iBAT. Then the adipose‐specific Sirt3 knockout (Sirt3 AKO) mice were used to investigate the role of Sirt3 in ACar metabolism and thermogenesis of BAT and explore the underlying mechanism, and the results showed that Sirt3 AKO mice displayed defective ACar metabolism and thermogenesis in iBAT. Mechanically, Sirt3 regulated ACar metabolism via HIF1α‐PPARα signaling pathway to promote iBAT thermogenesis, and knockdown or inhibition of HIF1α ameliorated impaired ACar metabolism and thermogenesis of iBAT in the absence of Sirt3. Collectively, we propose that Sirt3 regulated ACar metabolism is critical in maintaining thermogenesis in BAT of aging mice, which can promote the development of anti‐aging intervention strategy.

## Graphical abstract

The response of aging mice to L‐carnitine induced activation of ACar metabolism in BAT requires Sirt3 expression. Mechanically, Sirt3 regulated ACar metabolism via HIF1α‐PPARα signaling pathway to promote BAT thermogenesis. Sirt3 regulated ACar metabolism is critical in improving age‐associated decline of BAT thermogenesis.

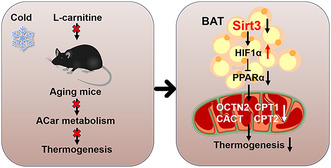

**Abbreviations**
ACar: acylcarnitine
Aks: adenylate kinases
BACs: brown adipocytes
BAT: brown adipose tissue
Bnip3: Bcl2/adenovirus E1B 19kDa interacting protein 3
CACT: carnitine‐acylcarnitine translocase
CPT1: carnitine palmitoyltransferase 1
CPT2: carnitine palmitoyltransferase 2
D‐Gal: D‐galactose
FAs: fatty acids
FFAs: free fatty acids
H&E: Hematoxylin and Eosin
HIF1α: hypoxia inducible factor 1α
iBAT: interscapular brown adipose tissue
LCar: L‐carnitine
LDs: lipid droplets
NMN: nicotinamide mononucleotide
OCTN2: organic cation transporter 2
PPARα: peroxisome proliferator activated receptor alpha
RER: respiratory exchange ratio
RNA‐seq: RNA sequencing
ROS: reactive oxygen species
RT: room temperature
SDR: short‐chain dehydrogenases/reductases
Sirt3 AKO: adipose‐specific Sirt3 knockout
Sirt3: Sirtuin 3
T3: tri‐iodothyronine
TG: triglyceride
THs: thyroid hormones
UCP1: uncoupling protein 1
Vegf: vascular endothelial growth factor
VO_2_
: oxygen consumption

## 1 INTRODUCTION

Aging is associated with metabolic decline (Gnad et al., 2020). A major health issue in the elderly is the loss of brown adipose tissue (BAT) activity, contributing to the impaired thermogenic response of the elderly (Wang, Song, et al., 2022). BAT has a powerful capacity to produce heat by uncoupling oxidative phosphorylation from ATP synthesis, which is conducted by uncoupling protein 1 (UCP1) (Zhang et al., 2018). However, the expression of UCP1 in BAT decline during aging, accompanied by the conversion of brown adipocytes (BACs) into white‐like phenotype (Gohlke et al., 2019). Further, the local microenvironment of BAT is altered during aging, resulting in the decrease of sympathetic innervation and changed endocrine signals that may inhibit thermogenesis during cold exposure (Mancini et al., 2021). As a thermogenic organ, BAT plays a crucial role in energy homeostasis and has been suggested as a potential target for treatment strategies of aging‐related metabolic disorders, such as diabetes and obesity (Gohlke et al., 2019). Therefore, improving BAT dysfunction of the elderly may be beneficial for metabolic health of aging.

Acylcarnitine (ACar) is arisen from the conjugation of L‐carnitine (LCar) with fatty acids (FAs) through carnitine palmitoyltransferase 1 (CPT1), which involved in various cellular energy metabolism pathways (Dambrova et al., 2022). ACar has been identified as a new energy substrate driving BAT thermogenesis (Simcox et al., 2017). During cold stimulation, circulating ACar levels are elevated, which are primary derived from the ACar synthesis in liver (Abumrad, 2017). Circulating ACar can be transported to BAT, which utilizes the ACar for thermogenesis to resist cold (Simcox et al., 2017). ACar are transported to mitochondrion through carnitine‐acylcarnitine translocase (CACT) in BAT (Dambrova et al., 2022). Once in the matrix, ACar are converted into carnitine and acyl‐CoAs through carnitine palmitoyltransferase 2 (CPT2) for subsequent β‐oxidation (Verkerke & Kajimura, 2021). LCar is a special amino acid that can assist the transport of long‐chain FAs from cytoplasm to mitochondrial matrix, which promotes intracellular lipid metabolism (Li & Zhao, 2021). It has been reported that LCar is necessary to maintain the phenotype and function of BAT in juvenile mice (Ozaki et al., 2011). Furthermore, a bolus injection of LCar has been demonstrated to reverse the cold‐sensitive phenotype of aging mice without the changes in thermogenic genes of BAT (Simcox et al., 2017). However, whether ACar metabolism underlies BAT thermogenesis decline with aging remain unclear.

Sirtuin 3 (Sirt3) belongs to nicotinamide adenine dinucleotide‐dependent deacetylase localized in mitochondria, which is essential for energy metabolism and cellular senescence (Covarrubias et al., 2021). Coincidently, BACs have dense mitochondria, leading to the high levels of Sirt3 in BAT. Sirt3 has been reported to be required for the expressions of BAT thermogenic genes of mice via regulating the acetylation status of UCP1 upstream genes in BAT (Sebaa et al., 2019). Sirt3 also plays an important role in the maintenance of BAT morphology and function (Gao et al., 2020). However, Porter et al. has reported that adipocyte Sirt3 is dispensable for maintaining mitochondrial function and systemic metabolism, and loss of Sirt3 dose not affect BAT function (Porter et al., 2018). Since the Sirt3 function in BAT is controversial, it is necessary to identify a novel role of Sirt3 in BAT during different physiological processes.

In our study, we administrated aging mice by LCar to explore the effects of chronic changes in circulating ACar levels on the cold sensitivity and interscapular BAT (iBAT) thermogenesis of aging mice. To our surprise, the cold sensitivity and iBAT thermogenesis of aging mice could not be improved by long term LCar feeding. We further investigated the reason why LCar has no effect on iBAT of aging mice. The results showed that Sirt3 might be a key regulator for the response of aging mice to LCar induced thermogenic activation, but Sirt3 expression was low in aging mice. We next generated adipose‐specific Sirt3 knockout (Sirt3 AKO) mice to explore the Sirt3 functions in iBAT of mice during cold exposure, and the results showed that Sirt3 AKO mice displayed defective ACar metabolism and thermogenesis in iBAT during cold exposure. Furthermore, we validated that Sirt3 modulated the transcription of ACar metabolism genes via regulating hypoxia inducible factor 1α (HIF1α)‐peroxisome proliferator activated receptor alpha (PPARα) signaling pathway to promote iBAT thermogenesis of mice during cold exposure. Finally, we found that lowering the level of HIF1α was required for the improvement of ACar metabolism and thermogenesis of iBAT in the absence of Sirt3.

## 2 RESULTS

### 2.1 The response to the activation of ACar metabolism is weakened during BAT aging

**FIGURE 1:**
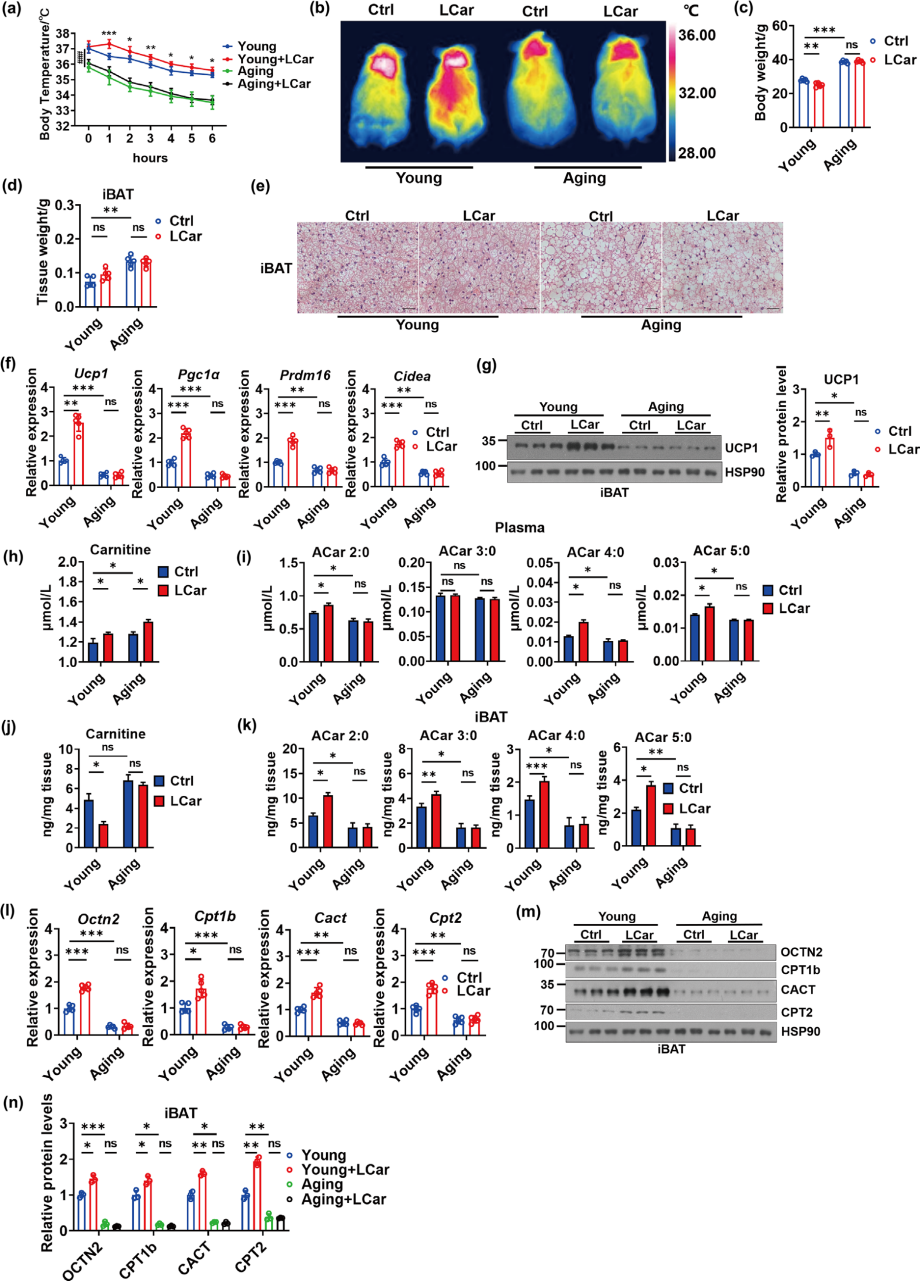
The response to the activation of ACar metabolism is weakened during BAT aging. (a) Core body temperature of young and aging mice treated with or without LCar during cold exposure (*n* = 5). *represents the significance between the young group and the young+LCar group, and ^#^represents the significance between the young group and the aging group at the starting point (0 hours) of cold exposure (b) Representative infrared images of dorsal skin temperature of young and aging mice treated with or without LCar after cold exposure. Body weight (c) and iBAT weight (d) of young and aging mice treated with or without LCar after cold exposure (*n* = 5). (e) Representative H&E staining of iBAT from young and aging mice treated with or without LCar after cold exposure (Scale bar, 50 μm). (f) Relative mRNA levels of *Ucp1*, *Pgc1α*, *Prdm16*, and *Cidea* in iBAT of young and aging mice treated with or without LCar after cold exposure (*n* = 5). (g) Western blots of UCP1 and HSP90 and relative protein level of UCP1 in iBAT of young and aging mice treated with or without LCar after cold exposure (*n* = 3). Plasma carnitine level (h) and short‐chain ACar levels (i) of young and aging mice treated with or without LCar after cold exposure (*n* = 3). iBAT carnitine level (j) and short‐chain ACar levels (k) of young and aging mice treated with or without LCar after cold exposure (*n* = 3). (l) Relative mRNA levels of *Octn2*, *Cpt1b*, *Cact*, and *Cpt2* in iBAT of young and aging mice treated with or without LCar after cold exposure (*n* = 5). (m) Western blots of OCTN2, CPT1b, CACT, CPT2, and HSP90 in iBAT of young and aging mice treated with or without LCar after cold exposure. (*n*) Relative protein levels of OCTN2, CPT1b, CACT, and CPT2 in iBAT of young and aging mice treated with or without LCar after cold exposure (*n* = 3). Data are presented as the mean ± SEM and n indicates the number of biologically independent experiments. *p < 0.05; **p < 0.01; ***p < 0.001; ^###^ *p* < 0.001 ns, not statistically significant (one‐way ANOVA).

To explore the effect of long term LCar feeding on the cold sensitivity and iBAT thermogenesis of young and aging mice, young mice (3 months old) and aging mice (18 months old) were oral administrated with LCar for 8 weeks. Then, mice were placed in 4°C for a 48 h cold challenge. The result showed that young mice received LCar displayed lower cold sensitivity and maintained higher body temperature during cold exposure, while we unexpectedly found that LCar failed to improve cold sensitivity of aging mice under acute cold challenge (Figure 1a). Consistently, the dorsal skin temperature and the rectal temperature after cold exposure for 48 h obtained the similar results (Figure 1b and S1a). Furthermore, LCar decreased the body weight of young mice after cold exposure(Figure 1c), while no change on the body weight of young mice after LCar feeding was occurred at room temperature (RT) (Figure S1b). Meanwhile, LCar had no effect on the body weight of aging mice both at RT and cold temperature. Young mice displayed higher oxygen consumption (VO_2_) and heat production at night in 16°C after LCar feeding, while the two indexes were still low in aging mice (Figure S1c). Meanwhile, LCar decreased the respiratory exchange ratio (RER) of young mice at night in 16°C, while aging mice displayed higher RER whether receiving LCar or not (Figure S1c). However, LCar had no effect on the VO_2_, RER, and heat production of young and aging mice at light (Figure S1d), and the differences between young and aging mice might attribute to aging‐associated dysfunction. The results suggested that LCar may upregulate basal metabolic rates and promote lipid utilization in young mice rather than in aging mice at night under mild cold ambient temperature. Moreover, LCar neither promoted nor improved the glucose tolerance and insulin sensitivity of young mice or aging mice at RT (Figure S1e,f). In addition, the blood glucose, serum triglyceride (TG) and free fatty acids (FFAs) were decreased in young mice by LCar after cold exposure, which were still remained high levels in aging mice after LCar feeding (Figure S1g–i). The iBAT weight of young mice was lower than that of aging mice, but LCar had no effect on iBAT weight of both young mice and aging mice after cold exposure (Figure 1d). The results of HE staining of iBAT indicated that more smaller lipid droplets (LDs) occurred in iBAT of young mice after LCar feeding during cold exposure, with big LDs exhibiting in iBAT of aging mice whether receiving LCar or not (Figure 1e), while LCar had no effect on the lipid accumulation in iBAT of mice at RT (Figure S1j). Meanwhile, young mice displayed higher expressions of thermogenic genes and proteins in iBAT after LCar feeding during cold exposure, while LCar was unable to increase expressions of thermogenic genes and proteins in iBAT of aging mice (Figure 1f,g). The expressions of thermogenic genes and proteins in iBAT were gradually reduced during aging at RT (Figure S1k, l), and LCar had no effect on the expressions of thermogenic genes in iBAT of both young and aging mice at RT (Figure S1m). Together, LCar could enhance iBAT thermogenesis of young mice after cold exposure, while aging mice might be insensitive to LCar, resulting in the unimproved iBAT thermogenesis after LCar feeding.

LCar promotes intracellular ACar metabolism, which is important for BAT thermogenesis. Therefore, we analyzed the ACar metabolism of young and aging mice after LCar feeding in response to cold. We found that young mice after LCar feeding displayed elevated level of plasma carnitine, which was further increased in aging mice after LCar feeding during cold exposure (Figure 1h). Meanwhile, the levels of short‐chain ACar, medium‐chain ACar, and long‐chain ACar in plasma of young mice were increased by LCar during cold exposure, but these ACar levels in plasma of aging mice were all lower than that of young mice and cannot be increased by LCar (Figure 1i and S1n,p). During cold exposure, the carnitine level of iBAT was reduced in young mice after LCar feeding, while aging mice with or without LCar feeding displayed higher carnitine levels in iBAT (Figure 1j). Moreover, the levels of short‐chain ACar, medium‐chain ACar, and long‐chain ACar of young mice and aging mice in iBAT displayed similar changing trends with plasma (Figure 1k and S1o,q). The results indicated that the ACar metabolism in iBAT might be abnormal in aging mice during cold exposure. Carnitine and short‐chain ACar can be transported into cells through organic cation transporter 2 (OCTN2), a cell‐surface transporter (Simcox et al., 2017; Wang et al., 2019). However, OCTN2 is not established as a transporter of medium‐chain and long‐chain ACar, and the transport mechanism of medium‐chain and long‐chain ACar into cells is still unknown. Therefore, the changes of OCTN2 in iBAT only reflect the transport of carnitine and short‐chain ACar to iBAT. During cold exposure, the gene and protein levels of OCTN2 in iBAT were increased in young mice after LCar feeding and were significantly decreased in aging mice with or without LCar feeding (Figure 1l–n), indicating that the transport of carnitine and short‐chain ACar from plasma to iBAT was promoted by LCar in young mice and was blocked in aging mice. This might partially explain why the plasma carnitine levels were higher in aging mice than in young mice after LCar feeding during cold exposure. And the changes of short‐chain ACar levels in iBAT might be partially attributed to the transport capacity of short‐chain ACar to iBAT in young and aging mice. During cold exposure, iBAT can also utilize carnitine to produce ACar for FFA oxidation. Therefore, although the levels of medium‐chain and long‐chain ACar were increased in plasma and iBAT of young mice with LCar feeding, it cannot be concluded that the transport of medium‐chain and long‐chain ACar to iBAT was increased. A part of medium‐chain and long‐chain ACar might be directly transformed from carnitine in iBAT. Thus, LCar feeding might influence the medium‐chain and long‐chain ACar levels in iBAT of young mice rather than aging mice during cold exposure. In addition, the gene and protein expressions of ACar metabolic enzymes (CPT1b, CACT, CPT2) in iBAT of young mice were increased by LCar, while the low expressions of these metabolic enzymes in iBAT were exhibited in aging mice with or without LCar feeding (Figure 1l–n). Besides, the expressions of ACar metabolic genes in iBAT were gradually reduced during aging at RT (Figure S1r), and LCar had no effect on the expressions of ACar metabolic genes in iBAT of both young and aging mice at RT (Figure S1s). The results suggested that LCar might promote the ACar metabolism in iBAT of young mice, leading to the decrease level of carnitine in iBAT during cold exposure, but the ACar levels in iBAT of young mice were increased. ACar are an intermediate of FFA oxidation, which led us to speculate that LCar might promote the metabolic flux of FFA oxidation in iBAT of young mice during cold exposure, resulting in the increase levels of ACar in iBAT. However, LCar might be unable to improve the ACar metabolism and the metabolic flux of FFA oxidation in iBAT of aging mice during cold exposure, leading to no improvement of carnitine and ACar levels in iBAT of aging mice after LCar feeding. Similarly, LCar had no effect on ACar metabolism in iBAT of young mice at RT. Based on the above data, we speculated that LCar‐mediated activation of BAT thermogenesis in young mice could be a cold‐inducible response. Consistently, LCar had no significant effect on UCP1 expression in BAT of young mice at RT, while the UCP1 expressions were increased (3.6‐fold change) under cold exposure and further increased by LCar (two‐fold change) at cold temperature (Figure S1t). Together, the highly response to the activation of ACar metabolism by LCar contributed to the enhancement of iBAT thermogenesis in young mice during cold exposure, while the response was weakened during BAT aging.

### 2.2 Adipose Sirt3 is required for the response to the activation of ACar metabolism in iBAT and plays an important role in iBAT thermogenesis

**FIGURE 2:**
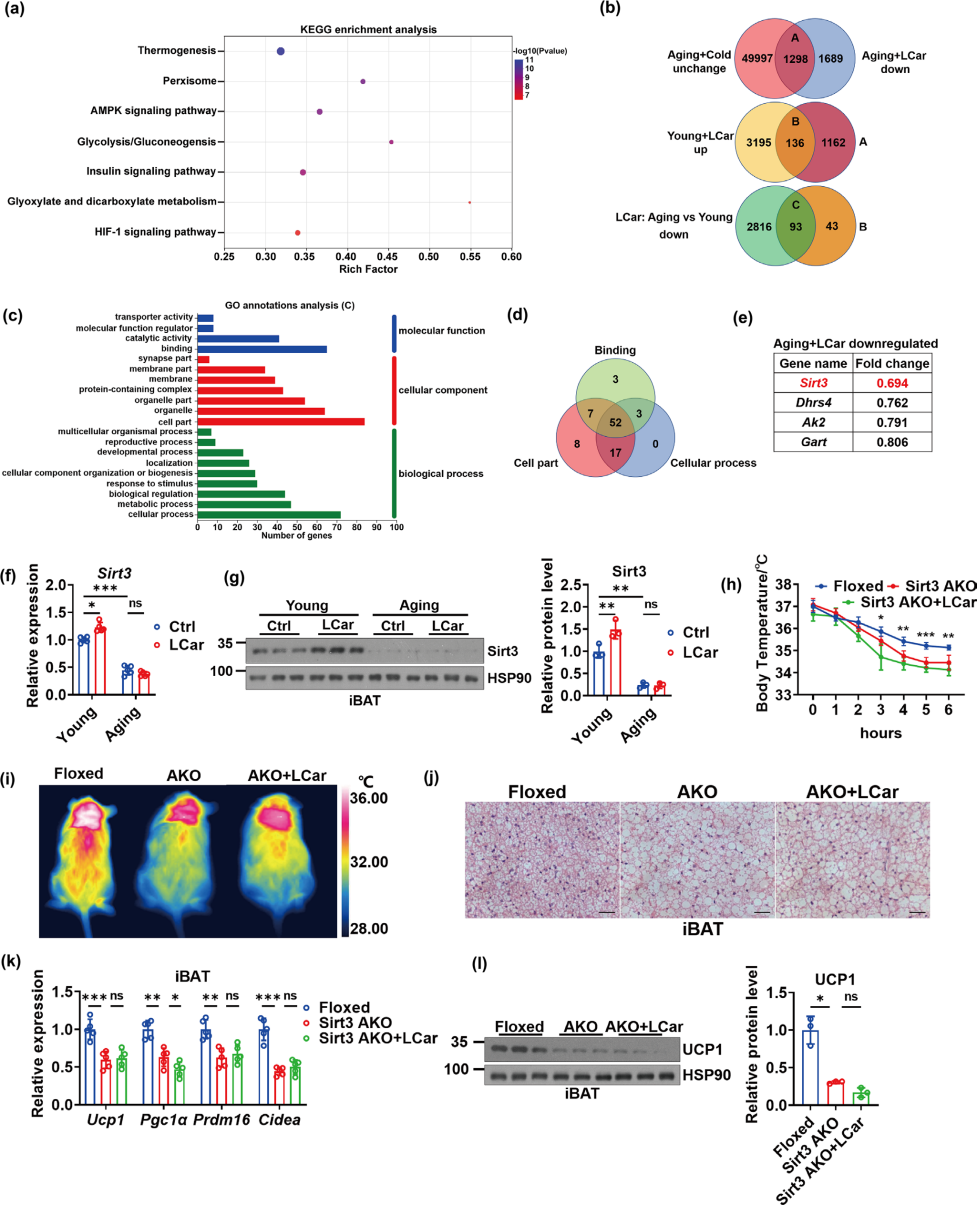
Adipose Sirt3 is required for the response to the activation of ACar metabolism in iBAT and plays an important role in iBAT thermogenesis. (a) KEGG enrichment analysis of iBAT genes in young and aging mice with LCar after cold exposure (*n* = 3). (b) Venn diagrams display the iBAT genes which were unchanged in aging mice after cold exposure and downregulated in aging mice after LCar administration, as labeled subset A (top); the iBAT genes which were upregulated in young mice after LCar administration and included in subset A of the top diagram, as labeled subset B (middle); the iBAT genes which were downregulated in aging mice compared to young mice after LCar administration and included in subset B of the middle diagram, as labeled subset C (bottom). (c) GO annotations analysis of iBAT genes in subset C. (d) Venn diagrams display the iBAT genes of the binding, cell part, and cellular process shared genes. (e) The fold changes of the iBAT genes involved in the metabolism of cofactors and vitamins pathway showed in d of aging mice after LCar feeding. (f) Relative mRNA levels of *Sirt3* in iBAT of young and aging mice treated with or without LCar after cold exposure (*n* = 5). (g) Western blots of Sirt3 and HSP90 and relative protein level of Sirt3 in iBAT of young and aging mice treated with or without LCar after cold exposure (*n* = 3). (h) Core body temperature of Sirt3 Floxed mice, Sirt3 AKO mice, and Sirt3 AKO mice treated with LCar during cold exposure (n = 5). (i) Representative infrared images of dorsal skin temperature of Sirt3 Floxed mice, Sirt3 AKO mice, and Sirt3 AKO mice treated with LCar after cold exposure. (j) Representative H&E staining of iBAT from Sirt3 Floxed mice, Sirt3 AKO mice, and Sirt3 AKO mice treated with LCar after cold exposure (Scale bar, 50 μm). (k) Relative mRNA levels of *Ucp1*, *Pgc1α*, *Prdm16*, and *Cidea* in iBAT of Sirt3 Floxed mice, Sirt3 AKO mice, and Sirt3 AKO mice treated with LCar after cold exposure (*n* = 5). (l) Western blots of UCP1 and HSP90 and relative protein level of UCP1 in iBAT of Sirt3 Floxed mice, Sirt3 AKO mice, and Sirt3 AKO mice treated with LCar after cold exposure (*n* = 3). Data are presented as the mean ± SEM and n indicates the number of biologically independent experiments. *p < 0.05; **p < 0.01; ***p < 0.001; ns, not statistically significant (one‐way ANOVA).

To further explore the molecular mechanism underlying the weak response to the activation of ACar metabolism in iBAT during aging, we performed RNA sequencing (RNA‐seq) analysis on iBAT of young and aging mice treated with or without LCar. The KEGG enrichment analysis showed that after LCar feeding, most of the genes that expressed lower in aging mice compared to young mice were associated with thermogenesis (Figure 2a), and the expression levels of these genes in the four groups of mice reflected the different effects of LCar on iBAT thermogenesis in young and aging mice (Figure S2a). We next performed the Venn analysis of the RNA‐seq results. Firstly, by overlapping the unchanged genes in iBAT of aging mice after cold exposure and the downregulated genes in iBAT of aging mice after LCar feeding in response to cold, 1298 genes were selected as subset A (Figure 2b). Next, by overlapping the upregulated genes in iBAT of young mice after LCar feeding in response to cold and the genes of subset A, 136 genes were selected as subset B (Figure 2b). Finally, we overlapped the downregulated genes in iBAT of aging mice compared to young mice after LCar feeding in response to cold and the genes of subset B, and the 93 differentially expressed genes were selected as candidates that might be important for iBAT thermogenesis of aging mice (Figure 2b). Furthermore, we performed GO annotations analysis on these 93 genes, and the binding of molecular function, the cell part of cellular component, and the cellular process of biological process involved the most number of genes, respectively (Figure 2c). Then, 52 genes shared in the three functions were elected (Figure 2d). Among the 52 genes, the pathway of metabolism of cofactors and vitamins in the Metabolism category contained the most number genes (Figure S2b), including *Ak2*, *Dhrs4*, *Gart*, and *Sirt3*. Based on the fold changes of the four genes in aging mice after LCar feeding, we identified *Sirt3* as a key gene in the regulation of iBAT thermogenesis via ACar metabolism of mice in response to cold (Figure 2e). The Sirt3 expressions in iBAT were gradually decreased during aging (Figure S2c, d). After cold exposure, the gene and protein expressions of Sirt3 were increased in iBAT of young mice after LCar feeding and were downregulated in aging mice whether receiving LCar or not, as well as the Sirt3 activity (Figure 2f,g and S2e). However, LCar had no effect on the Sirt3 expression in iBAT of both young and aging mice at RT (Figure S2f). Therefore, Sirt3 might be required for the response to the activation of ACar metabolism in iBAT of mice during cold exposure.

To investigate a novel role of Sirt3 in iBAT, we generated Sirt3 AKO mice, and the expression levels of Sirt3 were tested to verify the knockout efficiency (Figure S2g, h). The core body temperature of Sirt3 AKO mice significantly dropped during cold exposure (Figure 2h), and the dorsal skin temperature and the rectal temperature after cold exposure for 48 h obtained the similar results (Figure 2i and S2i), indicating that Sirt3 AKO mice had impaired iBAT thermogenesis. Sirt3 AKO mice displayed heavier body weight (Figure S2j), lower VO_2_ and heat production, higher RER (Figure S2k), higher blood glucose and serum TG (Figure S2l, m), and lower serum FFAs (Figure S2n) than the littermate control mice. The results indicated that low energy expenditure and disordered blood glucose and lipid levels occurred in Sirt3 AKO mice. However, LCar could not improve these abnormal indicators of Sirt3 AKO mice. Furthermore, the iBAT weight were increased in Sirt3 AKO mice compared with the littermate control mice (Figure S2o), with more larger LDs and cell size in iBAT during cold exposure (Figure 2j), indicating more lipid accumulation in iBAT of Sirt3 AKO mice. Meanwhile, the expression levels of thermogenic genes and proteins in iBAT of Sirt3 AKO mice were decreased (Figure 2k,l). LCar could not improve the thermogenic phenotype in iBAT of Sirt3 AKO mice. Collectively, these data suggested that Sirt3 might be crucial to iBAT thermogenesis during cold exposure, which prompted us to further investigate the specific role of Sirt3 in ACar metabolism of iBAT.

### 2.3 Both Sirt3 AKO and inhibition of OCTN2 cause abnormal ACar metabolism in iBAT

**FIGURE 3:**
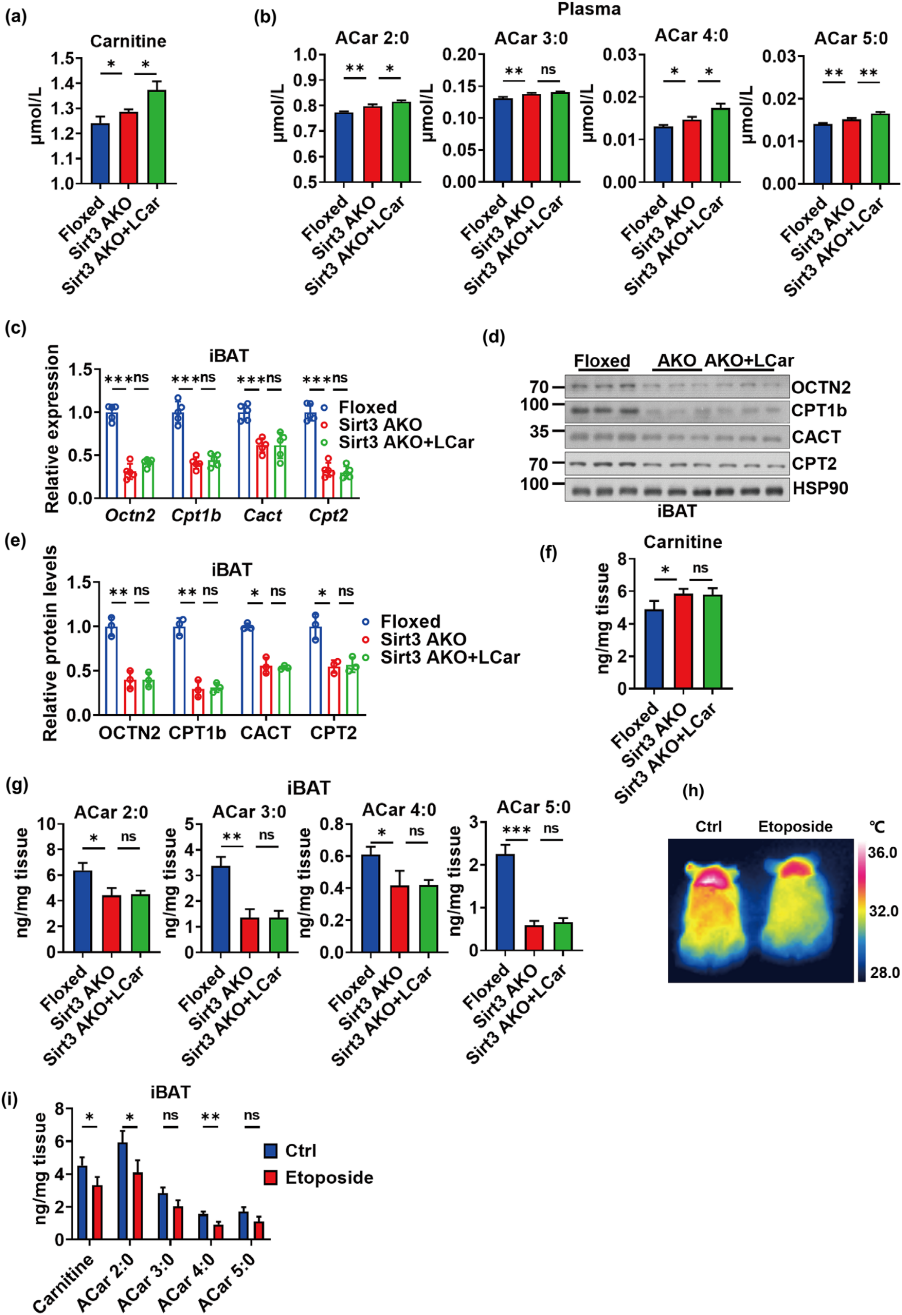
Both Sirt3 AKO and inhibition of OCTN2 cause abnormal ACar metabolism in iBAT. (a) Plasma carnitine level of Sirt3 Floxed mice, Sirt3 AKO mice, and Sirt3 AKO mice treated with LCar after cold exposure (*n* = 3). (b) Plasma short‐chain ACar levels of Sirt3 Floxed mice, Sirt3 AKO mice, and Sirt3 AKO mice treated with LCar after cold exposure (*n* = 3). (c) Relative mRNA levels of *Octn2*, *Cpt1b*, *Cact*, and *Cpt2* in iBAT of Sirt3 Floxed mice, Sirt3 AKO mice, and Sirt3 AKO mice treated with LCar after cold exposure (*n* = 5). (d) Western blots of OCTN2, CPT1b, CACT, CPT2, and HSP90 in iBAT of Sirt3 Floxed mice, Sirt3 AKO mice, and Sirt3 AKO mice treated with LCar after cold exposure. (e) Relative protein levels of OCTN2, CPT1b, CACT, and CPT2 in iBAT of Sirt3 Floxed mice, Sirt3 AKO mice, and Sirt3 AKO mice treated with LCar after cold exposure (*n* = 3). iBAT carnitine level (f) and short‐chain ACar levels (g) of Sirt3 Floxed mice, Sirt3 AKO mice, and Sirt3 AKO mice treated with LCar after cold exposure (*n* = 3). (h) Representative infrared images of dorsal skin temperature of young mice treated with or without etoposide. (i) iBAT carnitine and short‐chain ACar levels of young mice treated with or without etoposide after cold exposure (*n* = 3). Data are presented as the mean ± SEM and n indicates the number of biologically independent experiments. *p < 0.05; **p < 0.01; ***p < 0.001; ns, not statistically significant (Student's *t*‐test or one‐way ANOVA).

We analyzed the ACar metabolism of iBAT in Sirt3 AKO mice in response to cold. The results showed that the carnitine and short‐chain ACar levels in plasma of Sirt3 AKO mice were increased during cold exposure and further increased by LCar (Figure 3a,b). The results might be partially attributed to the decrease in the transport of carnitine and short‐chain ACar to iBAT characterized by the downregulation of OCTN2 expressions in iBAT of Sirt3 AKO mice (Figure 3c–e), leading to the accumulation of carnitine and short‐chain ACar in plasma during cold exposure. Moreover, the gene and protein expressions of ACar metabolic enzymes in iBAT were decreased in Sirt3 AKO mice whether received LCar or not during cold exposure (Figure 3c–e). Therefore, the ACar metabolic capacity in iBAT of Sirt3 AKO mice was reduced, resulting in the accumulation of carnitine and the decrease of short‐chain ACar transformed from carnitine in iBAT (Figure 3f,g). Apparently, the carnitine and short‐chain ACar levels in iBAT might be partially affected by the transport of carnitine and short‐chain ACar to iBAT. Consistently, the medium‐chain and long‐chain ACar levels in plasma and iBAT of Sirt3 AKO mice were similar to the short‐chain ACar levels (Figure S3a, b). Together, the ACar metabolism and the metabolic flux of FFA oxidation in iBAT of Sirt3 AKO mice might be downregulated, and the enhancement effects of LCar on iBAT thermogenesis were vanished in Sirt3 AKO mice. Our data suggested that Sirt3 might be important for ACar metabolism of iBAT, determining the supply and utilization of energy substrate for iBAT thermogenesis.

Furthermore, thyroid hormones (THs), especially tri‐iodothyronine (T3), play an important role in inducing thermogenesis during cold exposure (Bianco & McAninch, 2013). Therefore, we wonder whether T3 was involved in Sirt3‐regulated ACar metabolism and iBAT thermogenesis. We measured T3 levels in Sirt3 AKO mice. The results showed that the serum T3 levels were unchanged in the Sirt3 AKO mice compared with the Floxed mice after cold exposure, and LCar administration had no significant effect on serum T3 levels of Sirt3 AKO mice (Figure S3c). Our results suggested that adipose Sirt3 might not influence the systemic T3 homoeostasis, and T3 might not involved in the regulation of adipose Sirt3 on ACar metabolism and iBAT thermogenesis. To further understand whether T3 could participate in Sirt3‐dependent metabolic regulation of ACar in iBAT, we conducted experiments in vitro in BACs. The results showed that mature BACs incubated with 10 nM T3 for 24 h increased the expressions of Sirt3 and thermogenic genes (*Ucp1*, *Pgc1α*, *Prdm16*, and *Cidea*), as well as the genes involved in ACar metabolism (*Octn2*, *Cpt1b*, *Cact*, and *Cpt2*) (Figure S3d, e). Sirt3 gene silencing by siRNA significantly upregulated the expressions of genes involved in thermogenesis and ACar metabolism of BACs, while T3 treatment slightly increased these gene expressions in siSirt3 treated BACs except Sirt3 (Figure S3d, e). The results suggested that Sirt3‐regulated ACar metabolism and iBAT thermogenesis might be independent from T3. Together, T3 might not participate in Sirt3‐regulated ACar metabolism and iBAT thermogenesis, and T3‐dependent carnitine utilization and Sirt3‐dependent ACar metabolism might be two different stand‐alone pathways, jointly regulating iBAT thermogenesis.

In addition, to verify the contribution of OCTN2 in ACar metabolism and thermogenesis of iBAT, etoposide, a OCTN2 inhibitor, was used to inhibit OCTN2‐mediated transport of carnitine and short‐chain ACar (Hu et al., 2012). A single bolus of etoposide at a nontoxic dose of 10 mg/kg was administrated to wild type mice through intraperitoneal injection (Hu et al., 2012), and then the mice were exposed to cold for 48 h. The body temperature of mice after cold exposure for 48 h was decreased by etoposide (Figure 3h and S3f), and the Hematoxylin and Eosin (H&E) staining images showed more lipid accumulation in iBAT of mice administrated by etoposide (Figure S3g). The results indicated that etoposide‐mediated inhibition of OCTN2 might attenuate the iBAT thermogenesis. Moreover, etoposide treatment slightly reduced the carnitine and ACar levels in iBAT of mice during cold exposure (Figure 3i and S3h), indicating that the inhibition of OCTN2 not only reduced the transport of carnitine and short‐chain ACar to iBAT but also partially influenced the medium‐chain and long‐chain ACar levels in iBAT. Although we focused on the effect of etoposide on iBAT of mice, the impact of etoposide on other tissues could not be ignored, and the decrease of body temperature of mice after cold exposure might be a broad regulation by many tissues. Therefore, OCTN2 might play an essential role in the supply of energy substrate for iBAT thermogenesis.

### 2.4 Sirt3 modulates ACar metabolism in iBAT via regulating HIF1α‐PPARα pathway

To investigate the molecular mechanism underlying the regulation of Sirt3 on ACar metabolism in iBAT, we explored the downstream factors of Sirt3 and the upstream factors of genes involved in ACar metabolism (*Octn2*, *Cpt1b*, *Cact*, *Cpt2*). PPARα is proved to be a transcription factor of *Octn2*, *Cpt1b*, *Cact*, and *Cpt2* (Rolver et al., 2023; Wen et al., 2010; Zhao et al., 2021), prompting us to detect the expression of PPARα. The results showed that the expression levels of PPARα in iBAT of Sirt3 AKO mice were decreased, and LCar was unable to improve the decline (Figure S4a, b). Moreover, the expression levels of PPARα in iBAT of aging mice were lower than that of young mice after cold exposure, and D‐galactose (D‐Gal) stimulated BACs also displayed low PPARα expression (Figure S4c–e). Therefore, the results preliminarily demonstrated that Sirt3 might affect the transcription of ACar metabolism genes via regulating PPARα. Next, we determined to investigate the molecular connection between Sirt3 and PPARα. Sirt3 deficiency can cause the increase of cellar reactive oxygen species (ROS), which activates HIF1α and promotes its protein expression (Finley et al., 2011). PPARα is a downstream target of HIF1α, and the activation of HIF1α inhibits the PPARα expression (Xu et al., 2022). Therefore, we focused on the HIF1α‐PPARα signaling pathway in iBAT to confirm whether Sirt3 regulates ACar metabolism through this pathway. We firstly measured the ROS levels in the conditions of Sirt3 deficiency and aging, and the results showed the high relative ROS levels of BACs in Sirt3 AKO mice and aging mice as well as the D‐Gal stimulated BACs in vitro (Figure S4f, g). We next test the expression levels of HIF1α and its downstream target genes (vascular endothelial growth factor, *Vegf*; Bcl2/adenovirus E1B 19 kDa interacting protein 3, *Bnip3*) in iBAT. The mRNA levels of *Vegf* and *Bnip3* were increased in iBAT of Sirt3 AKO mice and aging mice after cold exposure as well as the protein levels of HIF1α, and the experiment in D‐Gal stimulated BACs in vitro showed the similar variations (Figure S4h–k). In addition, the gene expressions of *Vegf*, *Bnip3*, and *PPARα* in iBAT were gradually decreased during aging (Figure S4l), and LCar also had no effect on these gene expressions in iBAT of both young and aging mice at RT (Figure S4m). Together, Sirt3 could influence HIF1α‐PPARα signaling pathway in iBAT.

**FIGURE 4:**
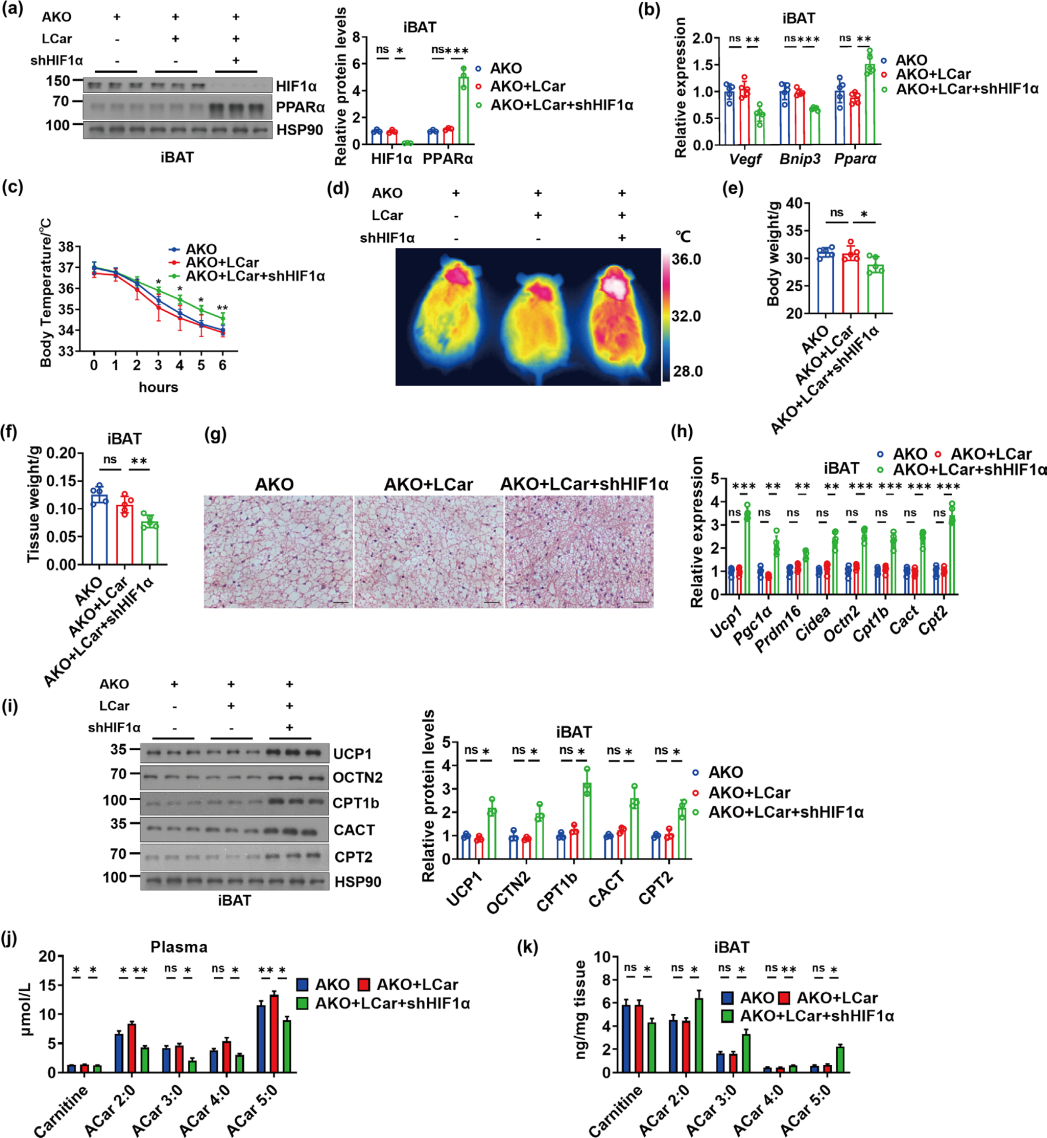
Sirt3 modulates ACar metabolism in iBAT via regulating HIF1α‐PPARα pathway. (a) Western blots of HIF1α, PPARα, and HSP90 and relative protein levels of HIF1α and PPARα in iBAT of Sirt3 AKO mice, Sirt3 AKO mice treated with LCar, and Sirt3 AKO mice treated with LCar and shHIF1α after cold exposure (*n* = 3). (b) Relative mRNA levels of *Vegf*, *Bnip3* and *Pparα* in iBAT of Sirt3 AKO mice, Sirt3 AKO mice treated with LCar, and Sirt3 AKO mice treated with LCar and shHIF1α after cold exposure (*n* = 5). (c) Core body temperature of Sirt3 AKO mice, Sirt3 AKO mice treated with LCar, and Sirt3 AKO mice treated with LCar and shHIF1α during cold exposure (*n* = 5). (d) Representative infrared images of dorsal skin temperature of Sirt3 AKO mice, Sirt3 AKO mice treated with LCar, and Sirt3 AKO mice treated with LCar and shHIF1α after cold exposure. Body weight (e) and iBAT weight (f) of Sirt3 AKO mice, Sirt3 AKO mice treated with LCar, and Sirt3 AKO mice treated with LCar and shHIF1α after cold exposure (*n* = 5). (g) Representative H&E staining of iBAT from Sirt3 AKO mice, Sirt3 AKO mice treated with LCar, and Sirt3 AKO mice treated with LCar and shHIF1α after cold exposure (Scale bar, 50 μm). (h) Relative mRNA levels of *Ucp1*, *Pgc1α*, *Prdm16*, *Cidea*, *Octn2*, *Cpt1b*, *Cact*, and *Cpt2* in iBAT of Sirt3 AKO mice, Sirt3 AKO mice treated with LCar, and Sirt3 AKO mice treated with LCar and shHIF1α after cold exposure (*n* = 5). (i) Western blots of UCP1, OCTN2, CPT1b, CACT, CPT2, and HSP90 and relative levels of these proteins in iBAT of Sirt3 AKO mice, Sirt3 AKO mice treated with LCar, and Sirt3 AKO mice treated with LCar and shHIF1α after cold exposure (*n* = 3). Carnitine and short‐chain ACar levels in plasma (j) and iBAT (k) of Sirt3 AKO mice, Sirt3 AKO mice treated with LCar, and Sirt3 AKO mice treated with LCar and shHIF1α after cold exposure (*n* = 3). Data are presented as the mean ± SEM and n indicates the number of biologically independent experiments. *p < 0.05; **p < 0.01; ***p < 0.001; ns, not statistically significant (one‐way ANOVA).

To investigate whether upregulated HIF1α is responsible for Sirt3 deficiency induced impairment of thermogenesis and ACar metabolism in iBAT, HIF1α was knocked down in Sirt3 AKO mice receiving LCar by in situ injection of shHIF1α in the iBAT pad. Mice were exposed to cold for 48 h at 3 weeks after injection. Significantly downregulated HIF1α protein level and gene expressions of *Vegf* and *Bnip3* were found in shHIF1α injected iBAT of Sirt3 AKO mice receiving LCar, with the upregulated gene and protein expressions of PPARα, after cold exposure (Figure 4a,b). Knockdown of HIF1α in iBAT increased body temperature of Sirt3 AKO mice receiving LCar during cold exposure, with the decrease of body weight, blood glucose, and serum TG and the increase of serum FFA (Figure 4c–e and S4n–q). Furthermore, knockdown of HIF1α decreased the iBAT weight and promote thermogenesis and ACar metabolism of iBAT in Sirt3 AKO mice receiving LCar, as demonstrated by histological analysis and transcriptional and protein levels of thermogenic and ACar metabolic genes (Figure 4f–i). Moreover, knockdown of HIF1α in iBAT decreased the carnitine and ACar levels in plasma of Sirt3 AKO mice receiving LCar after cold exposure, with the decrease of carnitine and increase of ACar levels in iBAT (Figure 4j,k and S4r‐u). The results indicated that knockdown of HIF1α in iBAT promoted the clearance of carnitine and ACar in plasma and the ACar metabolism and metabolic flux of FFA oxidation in iBAT of Sirt3 AKO mice receiving LCar during cold exposure. In addition, knockdown of HIF1α in BACs in vitro also enhanced the transcriptional and protein levels of PPARα, thermogenic genes, and ACar metabolic genes (Figure S4v, w). Collectively, these data demonstrated that Sirt3 promoted thermogenesis and ACar metabolism of iBAT via repressing HIF1α, and knockdown of HIF1α could ameliorate the impairment of thermogenesis and ACar metabolism of iBAT in the absence of Sirt3.

### 2.5 Lowering the level of HIF1α is required for the improvement of abnormal ACar metabolism of iBAT in the absence of Sirt3

**FIGURE 5:**
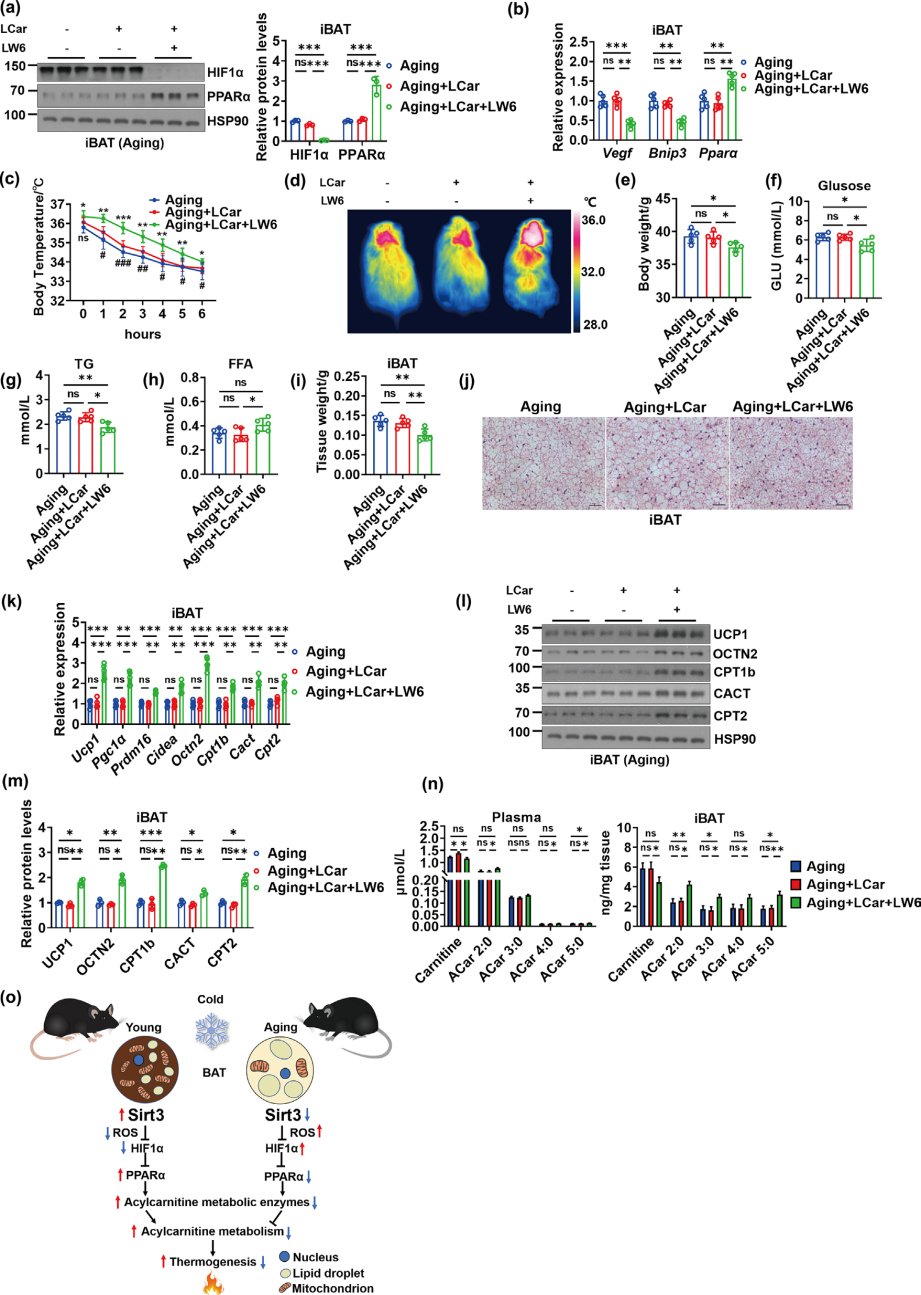
Lowering the level of HIF1α is required for the improvement of abnormal ACar metabolism of iBAT in the absence of Sirt3. (a) Western blots of PPARα, HIF1α, and HSP90 and relative protein levels of HIF1α and PPARα in iBAT of aging mice, aging mice treated with LCar, and aging mice treated with LCar and LW6 after cold exposure (*n* = 3). (b) Relative mRNA levels of *Vegf*, *Bnip3* and *Pparα* in iBAT of aging mice, aging mice treated with LCar, and aging mice treated with LCar and LW6 after cold exposure (*n* = 5). (c) Core body temperature of aging mice, aging mice treated with LCar, and aging mice treated with LCar and LW6 during cold exposure (*n* = 5). (d) Representative infrared images of dorsal skin temperature of aging mice, aging mice treated with LCar, and aging mice treated with LCar and LW6 after cold exposure. Body weight (e), blood glucose (f), serum TG (g), serum FFA (h), and iBAT weight (i) of aging mice, aging mice treated with LCar, and aging mice treated with LCar and LW6 after cold exposure (*n* = 5). (j) Representative H&E staining of iBAT from aging mice, aging mice treated with LCar, and aging mice treated with LCar and LW6 after cold exposure (Scale bar, 50 μm). (k) Relative mRNA levels of *Ucp1*, *Pgc1α*, *Prdm16*, *Cidea*, *Octn2*, *Cpt1b*, *Cact*, and *Cpt2* in iBAT of aging mice, aging mice treated with LCar, and aging mice treated with LCar and LW6 after cold exposure (*n* = 5). (l) Western blots of UCP1, OCTN2, CPT1b, CACT, CPT2, and HSP90 in iBAT of aging mice, aging mice treated with LCar, and aging mice treated with LCar and LW6 after cold exposure. (m) Relative levels of UCP1, OCTN2, CPT1b, CACT, and CPT2 iBAT of aging mice, aging mice treated with LCar, and aging mice treated with LCar and LW6 after cold exposure (*n* = 3). (n) Carnitine and short‐chain ACar levels in plasma (left) and iBAT (right) of aging mice, aging mice treated with LCar, and aging mice treated with LCar and LW6 after cold exposure (*n* = 3). (o) Schematic diagram of the working model upon young and aging. Data are presented as the mean ± SEM and n indicates the number of biologically independent experiments. *p < 0.05; **p < 0.01; ***p < 0.001; ns, not statistically significant (one‐way ANOVA).

To investigate whether exogenous inhibition of HIF1α ameliorates the impaired thermogenesis and ACar metabolism of iBAT in the absence of Sirt3, aging mice and Sirt3 AKO mice receiving LCar were administrated with LW6, a novel HIF1α inhibitor that promotes the degradation of HIF1α (Lee et al., 2010). Inhibition of HIF1α by LW6 significantly downregulated the HIF1α protein expressions and the gene expressions of *Vegf* and *Bnip3* in iBAT of both aging mice and Sirt3 AKO mice receiving LCar, with the PPARα up‐regulation in transcriptional and protein levels (Figure 5a,b and S5a, b). LW6 attenuated the cold sensitivity of aging and Sirt3 AKO mice receiving LCar during cold exposure, as demonstrated by the core body temperature and the infrared thermogram (Figure 5c,d and S5c, d). Inhibition of HIF1α by LW6 reduced the body weight, blood glucose, and serum TG levels and increased the serum FFA levels in both aging mice and Sirt3 AKO mice receiving LCar after cold exposure (Figure 5e–h and S5e–h). Furthermore, LW6 decreased the iBAT weight and promote thermogenesis and ACar metabolism of iBAT in both aging and Sirt3 AKO mice receiving LCar, as demonstrated by histological analysis and transcriptional and protein levels of thermogenic and ACar metabolic genes (Figure 5i–m and S5i‐l). Moreover, inhibition of HIF1α by LW6 decreased the carnitine levels in plasma and iBAT of aging mice receiving LCar after cold exposure, with the increase of ACar levels in both plasma and iBAT (Figure 5n and S5m–p). The results of LW6 on the carnitine and ACar levels in plasma and iBAT of Sirt3 AKO mice receiving LCar after cold exposure were consistent with that of shHIF1α (Figure S5q–u). Therefore, inhibition of HIF1α by LW6 might improve the ACar metabolism and the metabolic flux of FFA oxidation in iBAT of aging and Sirt3 AKO mice in response to cold. In addition, the inhibition of HIF1α by LW6 combined with LCar improved cold tolerance, blood glucose level, serum lipid levels, the lipid accumulation of iBAT, the expressions of thermogenic and ACar metabolic genes, and the ACar levels in plasma and iBAT of aging mice after cold exposure. Importantly, inhibition of HIF1α increased PPARα expressions in iBAT of aging mice with LCar administration, indicating the important role of HIF1α‐PPARα pathway in LCar‐induced activation of BAT thermogenesis in aging mice. Together, these data indicated that exogenous inhibition of HIF1α could ameliorate abnormal ACar metabolism of iBAT in the absence of Sirt3, improving iBAT thermogenesis in the case of different physiological and pathological status induced Sirt3 dysfunction. Importantly, LCar and LW6 administration could improve iBAT thermogenesis of aging mice via regulating ACar metabolism, suggesting that the Sirt3/HIF1α axis and ACar metabolism in BACs might be a potential therapeutic target for aging related metabolic disorder.

In addition, we also treated aging mice receiving LCar with nicotinamide mononucleotide (NMN), a well‐known Sirt3 activator (Wang, Sun, et al., 2022), to explore an effective nutritional intervention strategy via targeting Sirt3 to improve iBAT thermogenesis of aging mice. The results showed that NMN increased the body temperature of aging mice receiving LCar after cold exposure (Figure S6a), with the decrease of iBAT weight and lipid accumulation and the enhancement of gene expressions involved in thermogenesis and ACar metabolism (Figure S6b–d). Furthermore, the protein levels of Sirt3 in iBAT of aging mice were not improved by LCar treatment, while synergistic supplementation of LCar and NMN significantly increased the protein levels of Sirt3 in iBAT of aging mice (Figure S6e). Therefore, the improvement in body temperature, iBAT weight and lipid accumulation of iBAT, and the expressions of thermogenic and ACar metabolic genes in iBAT of aging mice under synergistic supplementation of LCar and NMN might attribute to the increase levels of Sirt3. Next, we used D‐galactose to induced senescence of BACs in vitro. The senescent BACs were treated with LCar, NMN, and the combination of NMN and LCar. The results showed that LCar had no effect on the expressions of Sirt3 and thermogenic genes of senescent BACs, while NMN treatment increased the expressions of Sirt3 and thermogenic genes, with the greater improvement by the combination of NMN and LCar (Figure S6f, g). The results suggested that the improvement in iBAT thermogenic function could be a result of NMN increasing Sirt3, and the greater improvement could be induced by a combination of NMN and LCar. Therefore, the combination of NMN and LCar could be another effective nutritional intervention strategy to maintain BAT thermogenesis of aging mice.

## 3 DISCUSSION

Aging, a well‐established risk factor for the decline in physical function, is closely associated with obesity, type 2 diabetes, cardiovascular disorders, cancer, and disorders (Lopez‐Otin et al., 2013). Efficient BAT activity is important for the maintenance of metabolic homeostasis and the reduced risk of type 2 diabetes (Lettieri‐Barbato & Aquilano, 2020), which makes BAT an ideal target to counteract aging and associated metabolic disorders. In this study, we provide evidence that aging‐associated Sirt3 decline contributes to the abnormal ACar metabolism of iBAT, impairing iBAT thermogenesis. Besides, we demonstrate a mechanistic link between Sirt3 and HIF1α, showing that Sirt3 inhibits HIF1α expression and manipulation of HIF1α level can ameliorate the impaired ACar metabolism and thermogenesis of iBAT in the absence of Sirt3.

Clear increases in thermogenesis and ACar metabolism of iBAT in young mice after cold exposure are shown after chronic LCar treatment. Nevertheless, LCar‐induced enhancement of iBAT thermogenesis and ACar metabolism was not appeared in young mice at RT. UCP1 expression in BAT determines the contribution of BAT to systemic metabolism (Ma et al., 2023). LCar had no significant effect on UCP1 expression in iBAT of young mice at RT, while the UCP1 expression was increased (3.6‐fold change) under cold exposure and further increased by LCar (2‐fold change) at cold temperature (Figure S1t). Therefore, the cold‐induced significant increase of UCP1 expressions in iBAT of young mice after LCar treatment might increase the contribution of BAT to systemic metabolism, contributing to the response of BAT to LCar appeared at cold temperature instead of RT. Moreover, based on a previous study (Simcox et al., 2017), serum ACar levels were significantly increased during acute cold exposure, and carnitine treatment induced significant increases of serum ACar levels in mice were occurred after cold exposure. Therefore, it could be calculated that carnitine‐mediated activation of BAT thermogenesis could be a cold‐inducible response, which was consistent with a cold‐inducible lncRNA266‐mediated browning and thermogenic program in white adipose tissue (Ma et al., 2023). Together, BAT and its thermogenic response are regulated by carnitine at cold temperature, which might attribute to the striking enhancement of UCP1 expression.

The metabolic effects of LCar supplementation vary from no effects to improvements in the burning capacity, either in muscle or BAT. In our study, LCar supplementation had no significant effects on BAT thermogenesis of aging mice. As to the discrepancy between our data here and others regarding role of LCar supplementation in the burning capacity of muscle or BAT, we speculate that tissue types, biological species, pathological states, or experimental conditions might be the reasons why different studies yield different results. LCar supplementation has been reported to have beneficial effects on muscle metabolism (Pereyra et al., 2020). The study focus on muscle, while we focus on BAT. The metabolic regulation of muscle and BAT are different. Furthermore, LCar has been reported to be essential for maintaining the function and morphology of BAT in the juvenile visceral steatosis mice, and the brown adipocyte differentiation and thermogenesis of goat was also required LCar (Ozaki et al., 2011; Wang et al., 2021). The model of juvenile visceral steatosis is different from aging in pathological state, and goat and mouse are different species. Moreover, the previous study has suggested that a bolus injection of LCar reverses the cold sensitivity of aging mice without the changes of iBAT thermogenic genes, which is merely dependent on the presence of iBAT (Simcox et al., 2017). Consistently, our study shows that long term LCar feeding has no effect on the expressions of iBAT thermogenic genes, but it fails to improve iBAT thermogenesis of aging mice during cold stimulation. The intervention method (intraperitoneal injection vs oral administration) and time (once vs. 8 weeks) are different. A single dose of LCar injection in the previous study might contribute to acute metabolic response, and the reversal of cold‐sensitive phenotype in aging mice might be an acute stress response. Long term LCar feeding in our study may be closer to the daily life, contributing to the beneficial metabolic remodeling in BAT of young mice and the failure of metabolic remodeling in BAT of aging mice due to the differential expression of Sirt3. Therefore, Sirt3 is important for the activation of ACar metabolism induced metabolic remodeling in iBAT of aging mice.

Sirt3 is required for maintenance of BAT functions. Our study demonstrates that Sirt3 is important for ACar metabolism in iBAT to maintain iBAT thermogenesis of aging mice during cold stimulation. Nevertheless, a previous study has reported that adipocyte Sirt3 is dispensable for maintaining mitochondrial function and systemic metabolism, and the metabolic phenotype of Sirt3 AKO mice in their study is similar to its littermate control mice upon regular chow diet or high fat diet at RT (Porter et al., 2018). In our study, the metabolic phenotype of Sirt3 AKO mice is displayed upon cold exposure, which is different from the previous study. Therefore, we speculate that Sirt3 may be important for iBAT thermogenesis in response to cold, while it is dispensable for iBAT upon normal condition or diet‐induced obesity. That is to say, Sirt3 is a temperature sensor gene, which varied with environment temperature. In addition, Porter et al. shows that the core body temperature of Sirt3 AKO mice is not changed during cold exposure for 2 h (Porter et al., 2018), which is consistent with our results. However, our results also show that the core body temperature of Sirt3 AKO mice is decreased after cold exposure for more than 2 h. Sirt3 is one of the key factor in counteracting aging (Covarrubias et al., 2021), which plays an important role in alleviating aging‐associated metabolic diseases on heart, kidney, neuron, and liver (Gomes et al., 2020; Zhang et al., 2021). Our study identifies a novel role of Sirt3 in maintaining BAT thermogenesis during aging.

In our study, *Dhrs4*, *Ak2*, and *Gart* were the significant changed genes in iBAT of aging mice treated with LCar after cold exposure (Figure 2e). Dhrs4 is a poorly characterized member of the short‐chain dehydrogenases/reductases (SDR) family (Persson & Kallberg, 2013). Dhrs4 is reported to have dual localization to peroxisomes and mitochondria and is dynamically phosphorylated and acetylated (Floyd et al., 2016). Dhrs4 interacts with other members of the SDR family, however, no direct function of Dhrs4 is known. Therefore, we speculated that Dhrs4 might not be related to ACar metabolism in BAT. Adenylate kinases (AKs) are phosphotransferases that catalyze the reversible conversion of adenosine triphosphate and adenosine monophosphate to adenosine diphosphate (ADP) (Dzeja & Terzic, 2009). As the only AK isoenzyme in the mitochondrial intermembrane space, AK2 plays an important role in mitochondrial energy metabolism (Dzeja & Terzic, 2009). However, there are no related study reporting the AK2 function in ACar metabolism, and thus it could be calculated that AK2 might not be closely related to ACar metabolism in BAT. Gart, as a key folate synthase in the purine de novo synthesis pathway, is reported to be expressed in the glia, fat body, and gut and positively regulates feeding behavior via cooperation and coordination (Tang et al., 2023). Gart was reported to play roles in energy homeostasis and lifespan. Similarly, there are no related study reporting the Gart function in ACar metabolism, and thus Gart might not be related to ACar metabolism in BAT. Sirt3 has been reported to be involved in the ACar metabolism, but the results are inconclusive and controversial (Hirschey et al., 2010; Sebaa et al., 2019). The role of Sirt3 in ACar metabolism and thermogenesis of BAT is not fully understood. Therefore, based on the RNA‐seq data and previous studies, we focused on the regulations of Sirt3 in ACar metabolism and thermogenesis of BAT during aging. An early study of Hirschey et al. suggested that Sirt3 whole body knock‐out mice had an accumulation of ACar in the plasma (Hirschey et al., 2010), while a recent study of Sebaa et al. reported the opposite results (Sebaa et al., 2019). In our study, the ACar levels in plasma of Sirt3 AKO mice are increased in response to cold (Figure 3b and S3a), which is consistent with the study of Hirschey et al. (2010). Furthermore, our study elucidates the dynamic changes of carnitine and ACar levels during their transportation and utilization in the absence of adipose Sirt3, highlighting the contribution of adipose Sirt3 in ACar metabolism of iBAT during cold stimulation. Importantly, our study clarifies a crucial role of Sirt3 in ACar metabolism to maintain iBAT thermogenesis in aging mice.

HIF1α is a special signaling mediator that can be regulated by oxygen (Agarwal et al., 2016). Under normoxia conditions, HIF1α is unstable due to ubiquitin‐mediated proteasomal degradation, but is stabilized in hypoxic conditions (Li et al., 2015). Hypoxia is associated with metabolic dysfunction, and HIF1α plays an essential role in the development of various metabolic diseases, such as obesity, inflammation, and cancer (Jun et al., 2017; Li et al., 2015). Adipose HIF1α promotes obesity, and HIF1α overexpression in adipose tissue inhibits BAT thermogenesis (Jun et al., 2017). However, adipose tissue‐specific inhibition of HIF1α was also demonstrated to induce obesity and inhibit BAT thermogenesis (Zhang et al., 2010). Therefore, the function of HIF1α in BAT is controversial. HIF1α destabilization can be regulated by Sirt3, while Sirt3 loss increases ROS production, resulting in HIF1α stabilization (Finley et al., 2011). Here, we provide evidence that Sirt3 deficiency causes HIF1α stabilization, and knockdown or inhibition of HIF1α in BAT ameliorates abnormal ACar metabolism in the absence of Sirt3. Importantly, we demonstrate that inhibition of HIF1α improves iBAT thermogenesis during aging.

In summary, this study report a function of Sirt3 as a sluice on ACar metabolism of iBAT and the effects of Sirt3‐HIF1α‐PPARα axis on iBAT thermogenesis in young and aging mice (Figure 5o). Our study identifies a novel role of Sirt3 in the regulation of BAT aging and provides theoretical basis for maintaining BAT thermogenesis of the elderly.

## 4 MATERIALS AND METHODS

### 4.1 Animals and treatments

All animal experiments were performed according to procedures approved by the Laboratory Animal Ethics Committee of Jiangnan University (permission no. JN.No20220615c0400930[247], JN.No20220515c0021201[157]) and complied with guidelines and regulations of the Guide for the Care and Use of Laboratory Animals. All mice were kept in a specific‐pathogen‐free animal facility with a 12 h dark/light cycle at a relative humidity of 55 ± 5% and constant temperature of 23 ± 2°C. All mice were given free access to a normal chow diet (AIN‐93G) and water. The young C57BL/6J male mice (3 months), middle‐aged male mice (10–12 months) and the aging male mice (18 months) were purchased from Charles River. Sirt3 AKO mice were generated using the Cre‐lox system by crossing Sirt3^f/f^ mice (Strain#031201) to adiponectin‐Cre^+/−^ mice (Strain#028020), which were all purchased from the Jackson Laboratory.

To investigate the improvement of LCar on thermogenesis of mice, we divided the young and aging mice into four groups: the young control group, the young‐LCar group, the aging control group, and the aging‐LCar group. LCar (Sigma‐Aldrich, Cat#64024B) was dissolved in 0.5% CMC‐Na aqueous solution, and the control group was given the same volume of CMC‐Na aqueous solution. The mice were oral administrated with LCar at 100 mg/kg/day for 8 weeks (Simcox et al., 2017). The Sirt3 AKO mice and Sirt3^f/f^ mice were born and housed in groups (5 mice per cage) until 12 weeks of age, which were divided into three groups: the Sirt3 Floxed mice (littermate control), the Sirt3 AKO mice, and the Sirt3 AKO mice received LCar (oral administration at 100 mg/kg/day for 8 weeks).

To investigate aging‐associated changes of mice, the young mice, middle‐aged mice, and aging mice were executed without any treatment.

For the knockdown of HIF1α in iBAT, 12 weeks old Sirt3 AKO mice receiving LCar for 5 weeks were injected orthotopically with 2.0 × 10^11^ vg of shHIF1α virus or shCtrl virus in the iBAT pad. The shHIF1α virus was synthetized in Shanghai Genechem Co., Ltd. After injection for 3 weeks, the mice were prepared for analysis. The mice were still oral administrated with LCar during the 3 weeks.

For the inhibition of HIF1α by LW6 (MedChemExpress, Cat#HY‐13671), both aging mice and Sirt3 AKO mice were divided into three groups: the control group, the LCar group, and the LCar+LW6 group. In the first 8 weeks, the control group were received CMC‐Na aqueous solution, and the LCar and LCar+LW6 groups were oral administrated with LCar at 100 mg/kg/day. Then, the LCar+LW6 group was administrated with LW6 by intraperitoneal injection at a dose of 20 mg/kg/day everyday for 7 days (Lee et al., 2010), with the other two groups intraperitoneal injecting the same volume of vehicle.

At the end of the treatment, mice were placed in a cold environment (4°C) for 48 h to conduct cold stimulation, and the rectal temperature was measured using a rectal thermometer (RWD Life Science) during cold exposure. For the measurement of energy expenditure, mice were individually placed into Comprehensive Laboratory Animal Monitoring System cages to measure the VO_2_, RER and heat production at a moderate cold temperature of 16°C. The iBAT temperature was measured after 48 h cold exposure, using an infrared camera (MAG 384 × 288; Magnity Technologies) according to previous publications (Liu et al., 2022). Mice were anaesthetized by intraperitoneal injection of isoflurane. The images were captured from the backs of the mice, which were displayed with the rainbow high contrast color palette. The blood glucose of mice was measured with a blood glucose monitor (Roche) after 48 h cold exposure.

### 4.2 Histology

The iBAT was isolated from mice and was fixed with 4% paraformaldehyde for 24 h and then embedded in paraffin. The embedded paraffin blocks were cut into 5 μm sections of entire block prepared using automatic constant temperature freezing microtome (Leica CM1950, Germany). The tissue sections were deparaffinized in xylene and were subsequently rehydrated using water and ethanol. The sections were stained with H&E after deparaffinization and rehydration. Finally, stained slides were sealed with resinene and visualized by inverted light microscopy (ZEISS Axio Vert A1, Germany).

### 4.3 RNA‐seq analysis

RNA‐seq analysis was performed in Shanghai Majorbio Biopharm Technology Limited Company. Total RNA was extracted from iBAT and quantified. Then, 5 μg RNA was used to construct the RNA‐seq transcriptome library according to the Illumina TruSeq RNA Sample Preparation Kit. After quantification by TBS380, the RNA‐seq sequencing library was constructed by the Illumina HiSeq X Ten (2 × 150 bp read length) after quantification by TBS380. The raw paired end reads were trimmed and quality controlled using SeqPrep and Sickle with default parameters. Next, clean reads were aligned to the reference genome with orientation mode by TopHat (version2.0.0) software. Furthermore, to identify differential expressed genes between two different groups, the expression level of each transcript was normalized according to the fragments per kilobase of exon per million mapped reads method.

### 4.4 ACar analysis

The measurement of ACar was performed as follows. Liquid chromatography–mass spectrometry based targeted metabolomics analysis was performed on a LC‐QTRAP 5500^+^‐MS/MS (Sciex, Concord, Ontario). LC separations were carried out on Kinetex C18 column (100 × 2.1 mm, particle size 2.6 μm, Phenomenex) on reverse phase mode for 24 min. Column temperature and flow rate were set to 40°C and 0.30 mL/min, respectively. The binary gradient system consisted of 0.1% formic acid in water (solvent A) and acetonitrile (solvent B). The linear gradient used for elution and equilibrating the initial gradient for subsequent runs was 5% B from 0 to 2 min, 5%–45% B from 2 to 5 min, 45%–100% B from 5 to 15 min, 100% B from 15 to 19 min, 100%–5% B from 19 to 20 min and 5% B from 20 to 24 min. The unlabeled and labeled Carnitine Standards Set B (Cambridge Isotope Laboratories Cat#NSK‐B‐US/Cat#NSK‐B) were used for the standard.

### 4.5 Statistical analysis

All values were presented as mean ± standard error mean (SEM). The Student's *t*‐test was applied for the assession of the difference between two groups, and one‐way ANOVA was used to analyze the difference between three groups. Statistical analysis was performed with GraphPad Prism 8.0 software. The significance was presented as * for *p* < 0.05, ** for *p* < 0.01, and *** for *p* < 0.001. All experiments were performed at least three times, and representative data were shown.

## AUTHOR CONTRIBUTIONS

*Conceptualization*: KZ and YL. *Animal and cell experimentation*: KZ and YW. *Data analysis*: KZ, YW, YS, LX, YW, CN, MF, and HQ. *Writing‐original draft*: KZ. *Writing, review, and editing*: KZ, YW, HY, LW, and YL. *Funding acquisition*: YW, HY, LW, and YL. All authors contributed to the article and approved the submitted version.

## FUNDING INFORMATION

This work was supported by grants from the National Natural Science Foundation of China (32,071,166, 92,357,303), the Earmarked Fund for China Agriculture Reasearch System (CARS‐08‐G19), the Young Elite Scientists Sponsorship Program by CAST (2020QNRC001), National Key Research and Development Program of China (2021YFA1100500, 2023YFA1801100), Fundamental Research Funds for the Central University (JUSRP221001), the Collaborative Innovation Center of Food Safety and Quality Control in Jiangsu Province, Jiangnan University (2022‐3‐1), Shanghai Municipal Science and Technology Major Project (GTP project), and General Program of Xuhui Health Commission of Shanghai (SHXH202211).

## CONFLICT OF INTEREST STATEMENT

The authors declare no conflict of interest.

## Supporting information


Data S1.

## Data Availability

The data supporting the conclusions of this investigation may be acquired from the corresponding author upon an appropriate demand. The RNA sequencing datasets have been deposited at the NCBI Sequence Read Archive and are accessible through accession number SRP441758.
