# Supplementary material for: Sirtuin 3 reinforces acylcarnitine metabolism and maintains thermogenesis in brown adipose tissue of aging mice
Source: Aging Cell. 2024 Sep 30;23(12):e14332. doi: 10.1111/acel.14332 (PMC11634729; doi:10.1111/acel.14332)
Supplement: Supplementary file 1 — Data S1. [file ACEL-23-e14332-s001.doc]

**Supplemental methods**

**1. Animal experiments**

For the etoposide treatment, C57BL/6J male mice aged at 12 weeks were treated with a single bolus of etoposide (Sigma-Aldrich, Cas#33419-42-0) dissolved in DMSO at a nontoxic dose of 10 mg/kg or DMSO by intraperitoneal injection (Hu et al., 2012), and then the mice were exposed to cold for 48 h.

To explore the nutritional intervention strategy via targeting Sirt3 to improve thermogenic capacity of aging mice, the aging mice were divided into three groups: the aging control group, the aging-LCar group, and the aging-LCar+NMN group. The LCar was also oral administrated to aging mice at a concentration of 100 mg/kg/day for 8 weeks. The NMN was dissolved in LCar solution at a final concentration of 250 mg/kg/day.

**2. Glucose Tolerance Test (GTT) and Insulin Tolerance Test (ITT)**

GTT and ITT were conducted before cold exposure. For GTT, the mice were injected intraperitoneally with 1 g/kg body weight of D-glucose (Sigma-Aldrich, Cat#G5767) after being fasted for 16 h. ITT was carried out on mice through intraperitoneal injection of regular human insulin (Novolin® 30R Penfill®, Novo Nordisk) at a dose of 0.75 U/kg body weight. Blood glucose levels were measured from the tail vein at 0, 15, 30, 60 and 120 min after injection using a blood glucose monitor (Roche).

**3. Biochemical analysis of serum**

The total TG levels in serum were measured according to the manufacturer’s protocols (Nanjing Jiancheng Bioengineering Institute, Cat#A110-1-1). Briefly, each serum sample was added to the well of 96-well plates and then mixed with the enzyme reagent provided in the assay kit. After incubation at 37 ℃ for 10 min, the absorbance of each well was measured by a Microplate reader (Thermo Fisher Scientific, Cat#1410101). The serum FFA was measured by a Free fatty Acids Con-tent Assay Kit (Solarbio, Cat#BC0590). Briefly, each serum sample was treated with n-Heptane: chloroform: absolute methanol (24:25:15) and centrifugated. Then, the supernatant was used to determine the FFA levels according to the manufacturer’s protocols. The T3 levels in serum were measured with ELISA kit according to the manufacturer’s protocols (Nanjing Jiancheng Bioengineering Institute, Cat#H224-1-1). Briefly, each serum sample was added to the well of ELISA plate and then mixed with the biotin antigen provided in the assay kit. After incubation at 37 ℃ for 30 min, the plate was washed for 5 times and then added with the avidin-HPR provided in the assay kit. After incubation at 37 ℃ for 30 min, the plate was washed for 5 times and then added with the chromogenic solution provided in the assay kit. After incubation at 37 ℃ for 10 min, the stop solution provided in the assay kit was added to each well to stop reaction. The absorbance of each well was measured at 450 nm by a Microplate reader (Thermo Fisher Scientific, Cat#1410101).

**4. RNA Isolation and qRT-PCR analysis**

Total RNA was extracted from different tissues of mice and BACs using RNAiso Plus (TaKaRa, Cat#9109) as described before (Li et al., 2018). All of RNA were quantified by an One Drop spectrophotometer. Complementary DNA was synthesized using Hifair® Ⅲ 1st Strand cDNA Synthesis SuperMix (Yeasen Biotech, Cat#11141ES60) according to the manufacture’s protocol. qRT-PCR was performed using Hieff® qPCR SYBR Green Master Mix (Yeasen Biotech, Cat#11203ES08) on a QuantStudio 3 RT-PCR system (Applied Biosystem, USA). As an internal control, β-actin was used to normalize the messenger RNA levels to determine the relative expressions of the target genes by using the 2−ΔΔCt method. Primer sequences used for qRT-PCR were shown in Table S1.

**5. Western blotting**

Samples were homogenized and extracted in RIPA lysis buffer (Beyotime, Cat#P0013B) with protease inhibitor cocktail and phosphatase inhibitor cocktail, following 10 min boiling and centrifugation to collect the supernatant for subsequent analyses. Protein concentration was determined using BCA Protein Quantification Kit (Vazyme, Cat#E112-01). Samples containing equal amounts of protein were resolved by 10% SDS-PAGE and subsequently transferred to polyvinylidene difluoride membranes. Membranes were incubated overnight at 4 ℃ with primary antibodies diluted in TBS containing 0.05% Tween and 5% BSA. After washing three times with TBST, the membranes were incubated with Peroxidase-AffiniPure Goat Anti-Rabbit/Mouse IgG (Jackson ImmunoResearch, Cat#111-035-003/Cat#115-005-003) at a dilution of 1:1000 for 1 h. Then, the membranes were washed another three times with TBST. Finally, the blots were visualized by ultrasensitive chemiluminescence reagent (Epizyme Biotech, Cat#SQ201) using an X-ray film processor (Delight, Suzhou, China). The primary antibodies are as follows: anti-Sirt3 (Cell Signaling Technology, 5490, 1:1000), anti-UCP1 (Abcam, ab10983, 1:10000,), anti-OCTN2 (Proteintech, 16331-1-AP, 1:1000), anti-CACT (Proteintech, 19363-1-AP, 1:4000), anti-CPT2 (Proteintech, 26555-1-AP, 1:2000), anti-CPT1b (Proteintech, 22170-1-AP, 1:1000), anti-PPARα (Santa Cruz Biotechnology, sc-398394, 1:1000), anti-HIF1α (ABclonal Cat#A17906, 1:1000), anti-HSP90 (Cell Signaling Technology, 4874, 1:2000).

**6. Acylcarnitine extraction**

The pretreatment of samples for acylcarnitine analysis was conducted as previously published with some modifications (Nemkov et al., 2023). Briefly, ~50 mg frozen tissue and 200 μL plasma were separately placed in the centrifuge tubes for extraction. Individual samples were added to 500 μL cold methanol and homogenized at a low temperature, and then the mixtures were sonicated for 15 min in ice water bath. Metabolite extracts were isolated by centrifugation at 12000g for 15 min at 4 ℃, and the supernatants were separated and completely dried by lyophilization. The dried samples were re-dissolved into 20% methanol for targeted metabolomics analysis.

**7. Sirt3 activity assay**

Sirt3 activity was measured using a Sirt3 Activity Assay Kit (Abcam, ab156067) following the manufacturer's protocol. In brief, firstly, the mitochondrial extract of iBAT was collected using the Tissue Mitochondria Isolation Kit (Beyotime Biotechnology, C3606) following the manufacturer's protocol. The mitochondrial extract was incubated with the Sirt3 assay buffer, Fluoro-Substrate Peptide, and NAD, and then co-incubated with Developer at 37 °C for 1 h. Fluorescent intensity was measured using a microplate fluorometer (Thermo Electron Corp) at 350 nm/450 nm.

**8.** **Cell culture**

The BACs were isolated from C57BL6J mice (Liu et al., 2022). The stromal vascular fraction (SVF)-derived preadipocytes from iBAT of newborn C57BL/6J mice were immortalized by SV40 retrovirus. Then, the cells were cultured in DMEM with 10% fetal bovine serum and 1% penicillin/streptomycin at 37 ℃ with 5% CO_2_. The number of cell passages was 3−4. To induce BACs differentiation, BACs were cultured in a growth medium with 5 μM dexamethasone, 0.1 μg/mL insulin, 0.125 mM indomethacin, 500 μM 3-isobutyl-1-methylxanthine, and 1 nM triiodothyronine for 48 h, and then it was replaced with another growth medium supplemented with 0.1 μg/mL insulin and 1 nM T3 for 4 days. The media were refreshed every other day. The differentiated mature adipocytes were used for further analysis. To induce cell senescence, BACs were incubated with 20 mg/mL D-Gal for 48 h and then were harvested for further analysis. For the knockdown of HIF1α, BACs were infected with 1 x 10^8^ vg/mL shHIF1α for 16 h. For the NMN intervention experiment, BACs were incubated with D-Gal for 48 h and then treated with 1 mM LCar (Sigma-Aldrich) or1 mM NMN or both LCar and NMN, respectively, for 24 h.

**9. Analysis of the contents of ROS**

The primary brown adipocytes were isolated from the iBAT of mice. The iBAT of mice were excised, minced and digested at 37 ℃ for 30 min. Cell suspensions were filtered through 100 μm filters and collected at 800g for 5 min. The isolated cells were seeded in culture dishes and cultured for the ROS detection of iBAT. The primary brown adipocytes and BACs were treated as indicated and incubated with fresh medium added containing 10 μM DCFH-DA (Sigma-Aldrich, Cat#D6883) at 37 ℃ for 30 min. The following protocols were conducted as previously published (Hao et al., 2017).

**References**

Hao, H., Cao, L., Jiang, C., Che, Y., Zhang, S., Takahashi, S., Wang, G., & Gonzalez, F. J. (2017). Farnesoid X receptor regulation of the NLRP3 inflammasome underlies cholestasis-associated sepsis. *Cell Metabolism*, *25*, 856-867. doi:10.1016/j.cmet.2017.03.007

Li, Y., Jiang, J., Liu, W., Wang, H., Zhao, L., Liu, S., Li, P., Zhang, S., Sun, C., Wu, Y., Yu, S., Li, X., Zhang, H., Qian, H., Zhang, D., Guo, F., Zhai, Q., Ding, Q., Wang, L., & Ying, H. (2018). microRNA-378 promotes autophagy and inhibits apoptosis in skeletal muscle. *Proceedings of the National Academy of Sciences, USA*, *115*, E10849-E10858. doi:10.1073/pnas.1803377115

Liu, S., Shen, S., Yan, Y., Sun, C., Lu, Z., Feng, H., Ma, Y., Tang, Z., Yu, J., Wu, Y., Gereben, B., Mohacsik, P., Fekete, C., Feng, X., Yuan, F., Guo, F., Hu, C., Shao, M., Gao, X., Zhao, L., Li, Y., Jiang, J., & Ying, H. (2022). Triiodothyronine (T3) promotes brown fat hyperplasia via thyroid hormone receptor alpha mediated adipocyte progenitor cell proliferation. *Nature Communications*, *13*, 3394. doi:10.1038/s41467-022-31154-1

Nemkov, T., Cendali, F., Stefanoni, D., Martinez, J. L., Hansen, K. C., San-Millan, I., & D'Alessandro, A. (2023). Metabolic signatures of performance in elite world tour professional male cyclists. *Sports Medicine*, *53*, 1651-1665. doi:10.1007/s40279-023-01846-9

**Supplemental Figures**


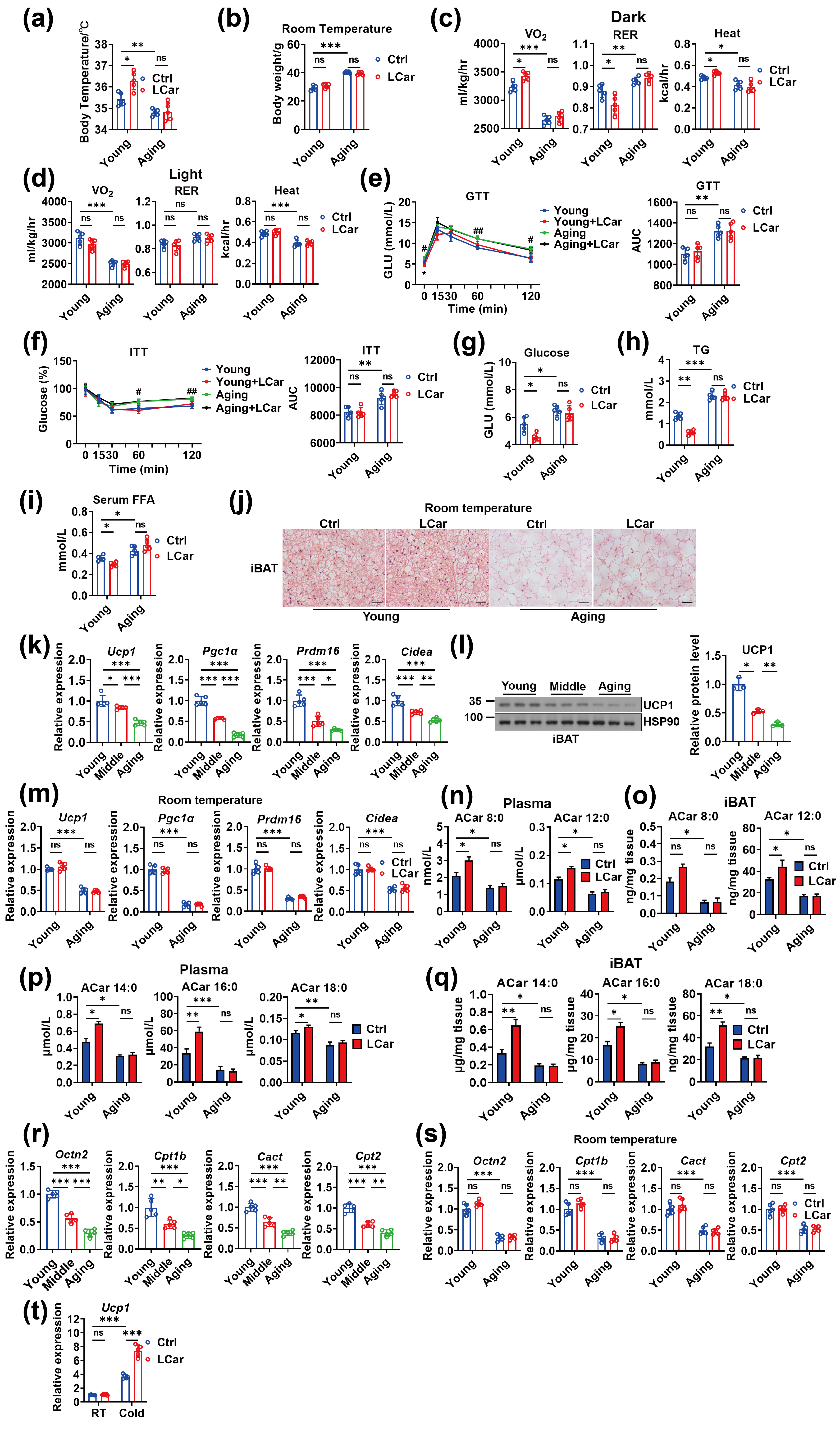


**Figure S1.** The response to the activation of acylcarnitine metabolism is weakened during BAT aging, related to Figure 1. (a) Core body temperature of young mice and aging mice treated with or without LCar after cold exposure for 48 h (n=5). (b) Body weight of young mice and aging mice treated with or without LCar at room temperature (n=5). (c) Average VO_2_, RER, and heat production of young mice and aging mice treated with or without LCar in 12 h dark at 16 °C (n=5). (d) Average VO_2_, RER, and heat production of young mice and aging mice treated with or without LCar in 12 h light at 16 °C (n=5). Glucose tolerance test (e) and insulin tolerance test (f) of young mice and aging mice treated with or without LCar at room temperature (n=5). In the left line chart, * represents the significance between the young group and the young+LCar group, and # represents the significance between the young group and the aging group. The blood glucose (g), serum TG (h), and FFAs (i) of young mice and aging mice treated with or without LCar after cold exposure (n=5). (j) Representative H&E staining of iBAT from young mice and aging mice treated with or without LCar at room temperature (Scale bar, 50 μm). (k) Relative mRNA levels of *Ucp1*, *Pgc1α*, *Prdm16*, and *Cidea* in iBAT of young mice, middle-aged mice, and aging mice at room temperature (n=5). (l) Western blots of UCP1 and HSP90 and relative protein level of UCP1 in iBAT of young mice, middle-aged mice, and aging mice at room temperature (n=3). (m) Relative mRNA levels of *Ucp1*, *Pgc1α*, *Prdm16*, and *Cidea* in iBAT of young mice and aging mice treated with or without LCar at room temperature (n=5). Medium-chain ACar levels of plasma (n) and iBAT (o) of young mice and aging mice treated with or without LCar after cold exposure (n=3). Long-chain ACar levels of plasma (p) and iBAT (q) of young mice and aging mice treated with or without LCar after cold exposure (n=3). (r) Relative mRNA levels of *Octn2*, *Cpt1b*, *Cact*, and *Cpt2* in iBAT of young mice, middle-aged mice, and aging mice at room temperature (n=5). (s) Relative mRNA levels of *Octn2*, *Cpt1b*, *Cact*, and *Cpt2* in iBAT of young mice and aging mice treated with or without LCar at room temperature (n=5). (t) Relative mRNA level of *Ucp1* in iBAT of young mice treated with or without LCar at room temperature or cold temperature (n=5). Data are presented as the mean ± SEM and n indicates the number of biologically independent experiments. **p* < 0.05; ***p* < 0.01; ****p* < 0.001; **#***p* < 0.05; **##***p* < 0.01; ns, not statistically significant (one-way ANOVA).


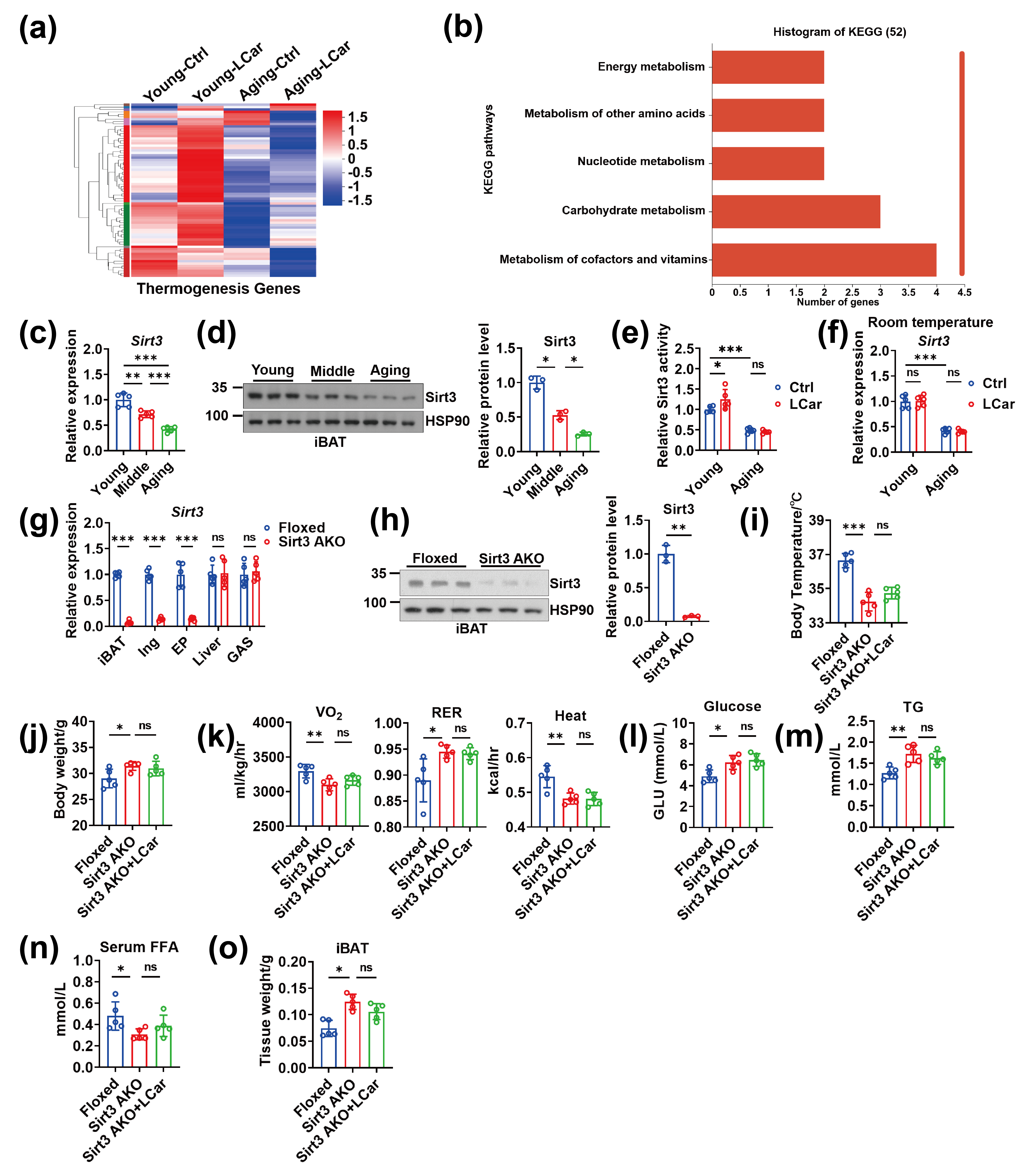


**Figure S2.** Adipose Sirt3 is required for the response to the activation of acylcarnitine metabolism in iBAT and plays an important role in iBAT thermogenesis, related to Figure 2. (a) Heatmap of thermogenic genes in iBAT of young mice and aging mice with or without LCar after cold exposure, related to Fig.2a. (b) KEGG pathway histogram of the genes in Fig.2d. (c) Relative mRNA level of *Sirt3* in iBAT of young mice, middle-aged mice, and aging mice at room temperature (n=5). (d) Western blots of Sirt3 and HSP90 and relative protein level of Sirt3 in iBAT of young mice, middle-aged mice, and aging mice at room temperature (n=3). (e) Relative Sirt3 activity in iBAT of young mice and aging mice treated with or without LCar after cold exposure (n=5). (f) Relative mRNA level of *Sirt3* in iBAT of young mice and aging mice treated with or without LCar at room temperature (n=5). (g) Relative mRNA levels of *Sirt3* in iBAT, inguinal adipose tissue (Ing), epididymal fat (EP), liver, and gastrocnemius (GAS) of Sirt3 Floxed and Sirt3 AKO mice (n=5). (h) Western blots of Sirt3 and HSP90 and relative protein level of Sirt3 in iBAT of Sirt3 Floxed and Sirt3 AKO mice. (i) Core body temperature of Sirt3 Floxed mice, Sirt3 AKO mice, and Sirt3 AKO mice treated with LCar after cold exposure for 48 h (n=5). (j) Body weight of Sirt3 Floxed mice, Sirt3 AKO mice, and Sirt3 AKO mice treated with LCar after cold exposure (n=5). (k) Average VO_2_, RER, and heat production of Sirt3 Floxed mice, Sirt3 AKO mice, and Sirt3 AKO mice treated with LCar at 16 °C (n=5). Blood glucose (l), serum TG (m), serum FFA (n), and iBAT weight (o) of Sirt3 Floxed mice, Sirt3 AKO mice, and Sirt3 AKO mice treated with LCar after cold exposure (n=5). Data are presented as the mean ± SEM and n indicates the number of biologically independent experiments. **p* < 0.05; ***p* < 0.01; ****p* < 0.001; ns, not statistically significant (Student’s t-test or one-way ANOVA).


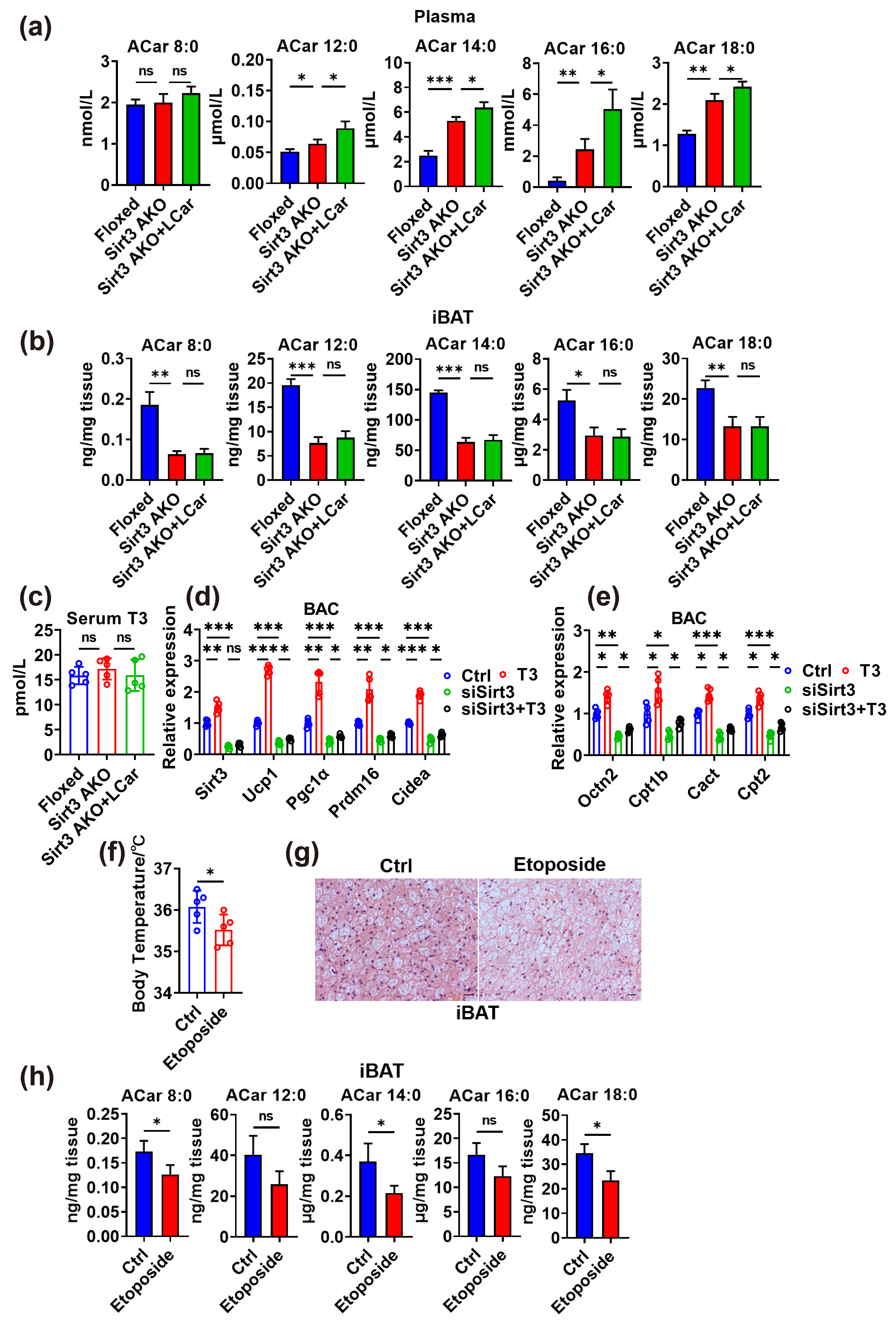


**Figure S3.** Both Sirt3 AKO and inhibition of OCTN2 cause abnormal acylcarnitine metabolism in iBAT, related to Figure 3. Medium-chain and long-chain ACar levels in plasma (a) and iBAT (b) of Sirt3 Floxed mice, Sirt3 AKO mice, and Sirt3 AKO mice treated with LCar after cold exposure (n=3). (c) Serum T3 level of Sirt3 Floxed mice, Sirt3 AKO mice, and Sirt3 AKO mice treated with LCar after cold exposure (n=5). (d) Relative mRNA levels of *Sirt3*, *Ucp1*, *Pgc1α*, *Prdm16*, and *Cidea* in siSirt3 infected BACs treated with or without T3 in vitro (n=5). (e) Relative mRNA levels of *Octn2*, *Cpt1b*, *Cact*, and *Cpt2* in siSirt3 infected BACs treated with or without T3 in vitro (n=5). (f) Core body temperature of young mice treated with or without etoposide after cold exposure for 48 h (n=5). (g) Representative H&E staining of iBAT from young mice treated with or without etoposide after cold exposure (Scale bar, 50 μm). (h) Medium-chain and long-chain ACar levels in iBAT of young mice treated with or without etoposide after cold exposure (n=3). Data are presented as the mean ± SEM and n indicates the number of biologically independent experiments. **p* < 0.05; ***p* < 0.01; ****p* < 0.001; ns, not statistically significant (Student’s t-test or one-way ANOVA).


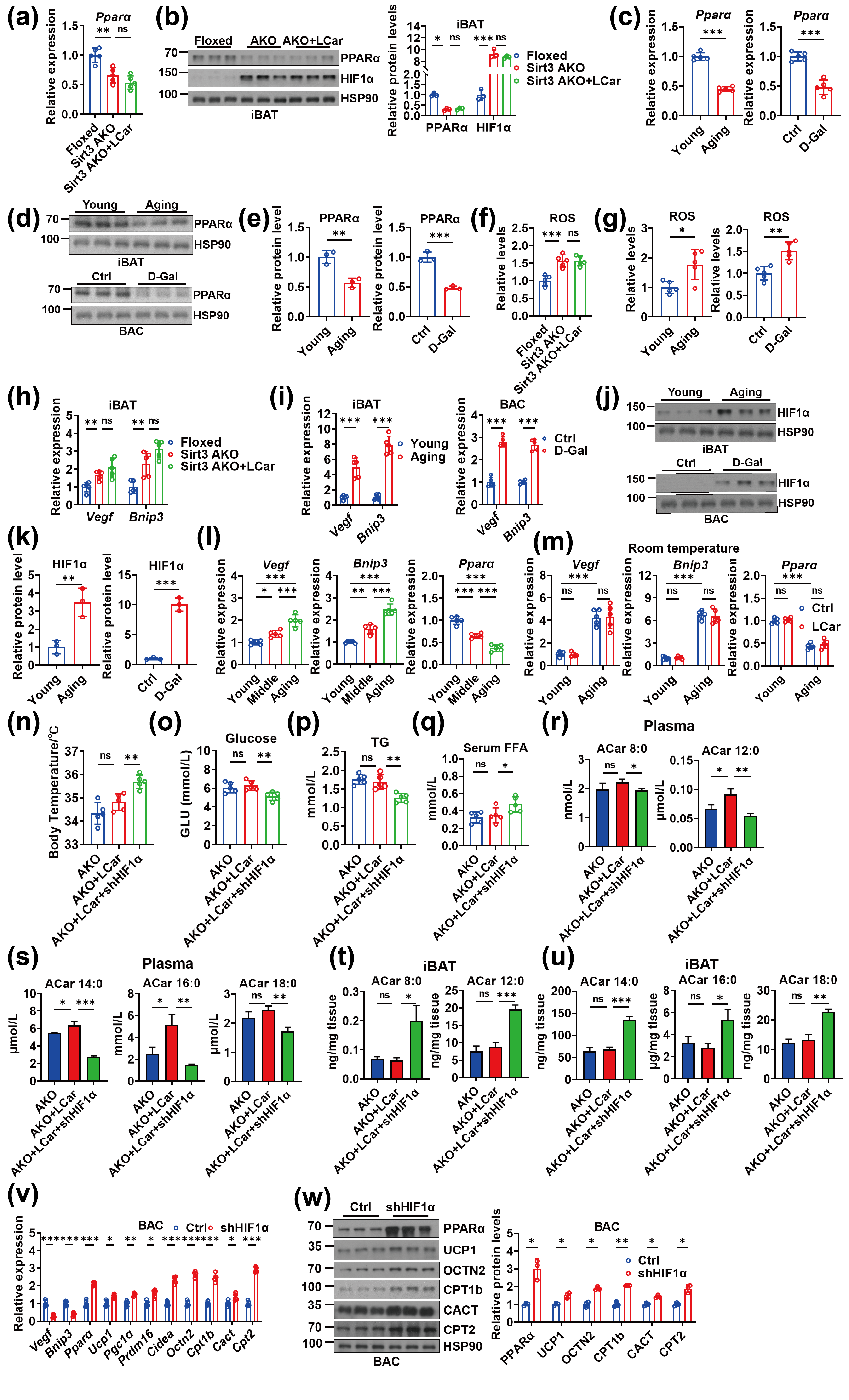


**Figure S4.** Sirt3 modulates acylcarnitine metabolism in iBAT via regulating HIF1α-PPARα pathway, related to Figure 4. (a) Relative mRNA levels of *Pparα* in iBAT of Sirt3 Floxed mice, Sirt3 AKO mice, and Sirt3 AKO mice treated with LCar after cold exposure (n=5). (b) Western blots of PPARα, HIF1α, and HSP90 and relative protein levels of HIF1α and PPARα in iBAT of Sirt3 Floxed mice, Sirt3 AKO mice, and Sirt3 AKO mice treated with LCar after cold exposure (n=3). (c) Relative mRNA levels of *Pparα* in iBAT of young and aging mice after cold exposure in vivo and D-Gal treated BACs in vitro (n=5). (d) Western blots of PPARα and HSP90 in iBAT of young and aging mice after cold exposure in vivo and D-Gal treated BACs in vitro. (e) Relative protein levels of PPARα in iBAT of young and aging mice after cold exposure in vivo and D-Gal treated BACs in vitro (n=3). (f) Relative ROS levels in iBAT of Sirt3 Floxed mice, Sirt3 AKO mice, and Sirt3 AKO mice treated with LCar after cold exposure (n=5). (g) Relative ROS levels in iBAT of young and aging mice after cold exposure in vivo and D-Gal treated BACs in vitro (n=5). (h) Relative mRNA levels of *Vegf* and *Bnip3* in iBAT of Sirt3 Floxed mice, Sirt3 AKO mice, and Sirt3 AKO mice treated with LCar after cold exposure (n=5). (i) Relative mRNA levels of *Vegf* and *Bnip3* in iBAT of young and aging mice after cold exposure and D-Gal treated BACs in vitro (n=5). (j) Western blots of HIF1α and HSP90 in iBAT of young and aging mice after cold exposure in vivo and D-Gal treated BACs in vitro. (k) Relative protein levels of HIF1α in iBAT of young and aging mice after cold exposure in vivo and D-Gal treated BACs in vitro (n=3). (l) Relative mRNA levels of *Vegf*, *Bnip3* and *Pparα* in iBAT of young mice, middle-aged mice, and aging mice at room temperature (n=5) (m) Relative mRNA levels of *Vegf*, *Bnip3* and *Pparα* in iBAT of young mice and aging mice treated with or without LCar at room temperature (n=5). (n) Core body temperature of Sirt3 AKO mice, Sirt3 AKO mice treated with LCar, and Sirt3 AKO mice treated with LCar and shHIF1α after cold exposure for 48 h (n=5). Blood glucose (o), serum TG (p), and FFA (q) of Sirt3 AKO mice, Sirt3 AKO mice treated with LCar, and Sirt3 AKO mice treated with LCar and shHIF1α after cold exposure (n=5). Medium-chain ACar levels (r) and long-chain ACar levels (s) in plasma of Sirt3 AKO mice, Sirt3 AKO mice treated with LCar, and Sirt3 AKO mice treated with LCar and shHIF1α after cold exposure (n=3). Medium-chain ACar levels (t) and long-chain ACar levels (u) in iBAT of Sirt3 AKO mice, Sirt3 AKO mice treated with LCar, and Sirt3 AKO mice treated with LCar and shHIF1α after cold exposure (n=3). (v) Relative mRNA levels of *Vegf*, *Bnip3*, *Pparα*, *Ucp1*, *Pgc1α*, *Prdm16*, *Cidea*, *Octn2*, *Cpt1b*, *Cact*, and *Cpt2* in shHIF1α infected BACs in vitro (n=5). (w) Western blots of PPARα, UCP1, OCTN2, CPT1b, CACT, CPT2, and HSP90 and relative levels of these proteins in shHIF1α infected BACs in vitro. Data are presented as the mean ± SEM and n indicates the number of biologically independent experiments. **p* < 0.05; ***p* < 0.01; ****p* < 0.001; ns, not statistically significant (Student’s t-test or one-way ANOVA).


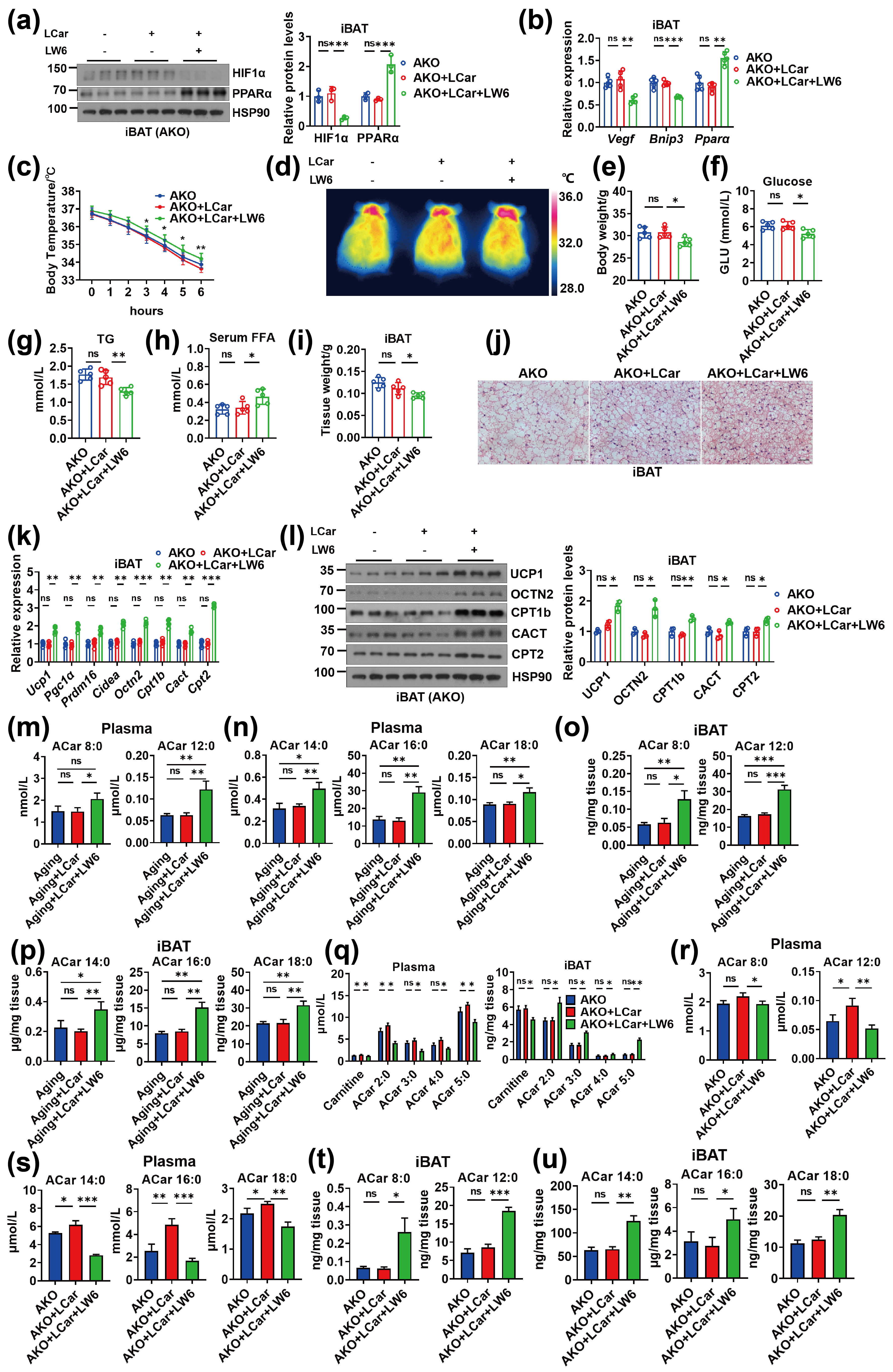


**Figure S5.** Lowering the level of HIF1α is required for the improvement of abnormal acylcarnitine metabolism of iBAT in the absence of Sirt3, related to Figure 5. (a) Western blots of PPARα, HIF1α, and HSP90 and relative protein levels of HIF1α and PPARα in iBAT of Sirt3 AKO mice, and Sirt3 AKO mice treated with LCar, and Sirt3 AKO mice treated with LCar and LW6 after cold exposure (n=3). (b) Relative mRNA levels of *Vegf*, *Bnip3*, and *Pparα* in iBAT of Sirt3 AKO mice, and Sirt3 AKO mice treated with LCar, and Sirt3 AKO mice treated with LCar and LW6 after cold exposure (n=5). (c) Core body temperature of Sirt3 AKO mice, and Sirt3 AKO mice treated with LCar, and Sirt3 AKO mice treated with LCar and LW6 during cold exposure (n=5). (d) Representative infrared images of dorsal skin temperature of Sirt3 AKO mice, and Sirt3 AKO mice treated with LCar, and Sirt3 AKO mice treated with LCar and LW6 after cold exposure. Body weight (e), blood glucose (f), serum TG (g), serum FFA (h), and iBAT weight (i) of Sirt3 AKO mice, and Sirt3 AKO mice treated with LCar, and Sirt3 AKO mice treated with LCar and LW6 after cold exposure (n=5). (j) Representative H&E staining of iBAT from Sirt3 AKO mice, and Sirt3 AKO mice treated with LCar, and Sirt3 AKO mice treated with LCar and LW6 after cold exposure (Scale bar, 50 μm). (k) Relative mRNA levels of *Ucp1*, *Pgc1α*, *Prdm16*, *Cidea*, *Octn2*, *Cpt1b*, *Cact*, and *Cpt2* in iBAT of Sirt3 AKO mice, and Sirt3 AKO mice treated with LCar, and Sirt3 AKO mice treated with LCar and LW6 after cold exposure (n=5). (l) Western blots of UCP1, OCTN2, CPT1b, CACT, CPT2, and HSP90 and relative levels of these proteins in iBAT of Sirt3 AKO mice, and Sirt3 AKO mice treated with LCar, and Sirt3 AKO mice treated with LCar and LW6 after cold exposure (n=3). Medium-chain ACar levels (m) and long-chain ACar levels (n) in plasma of aging mice, aging mice treated with LCar, and aging mice treated with LCar and LW6 after cold exposure (n=3). Medium-chain ACar levels (o) and long-chain ACar levels (p) in iBAT of aging mice, aging mice treated with LCar, and aging mice treated with LCar and LW6 after cold exposure (n=3). (q) Carnitine and short-chain ACar levels in plasma (left) and iBAT (right) of Sirt3 AKO mice, Sirt3 AKO mice treated with LCar, and Sirt3 AKO mice treated with LCar and LW6 after cold exposure (n=3). Medium-chain ACar levels (r) and long-chain ACar levels (s) in plasma of Sirt3 AKO mice, and Sirt3 AKO mice treated with LCar, and Sirt3 AKO mice treated with LCar and LW6 after cold exposure (n=3). Medium-chain ACar levels (t) and long-chain ACar levels (u) in iBAT of Sirt3 AKO mice, and Sirt3 AKO mice treated with LCar, and Sirt3 AKO mice treated with LCar and LW6 after cold exposure (n=3). Data are presented as the mean ± SEM and n indicates the number of biologically independent experiments. **p* < 0.05; ***p* < 0.01; ****p* < 0.001; ns, not statistically significant (one-way ANOVA).


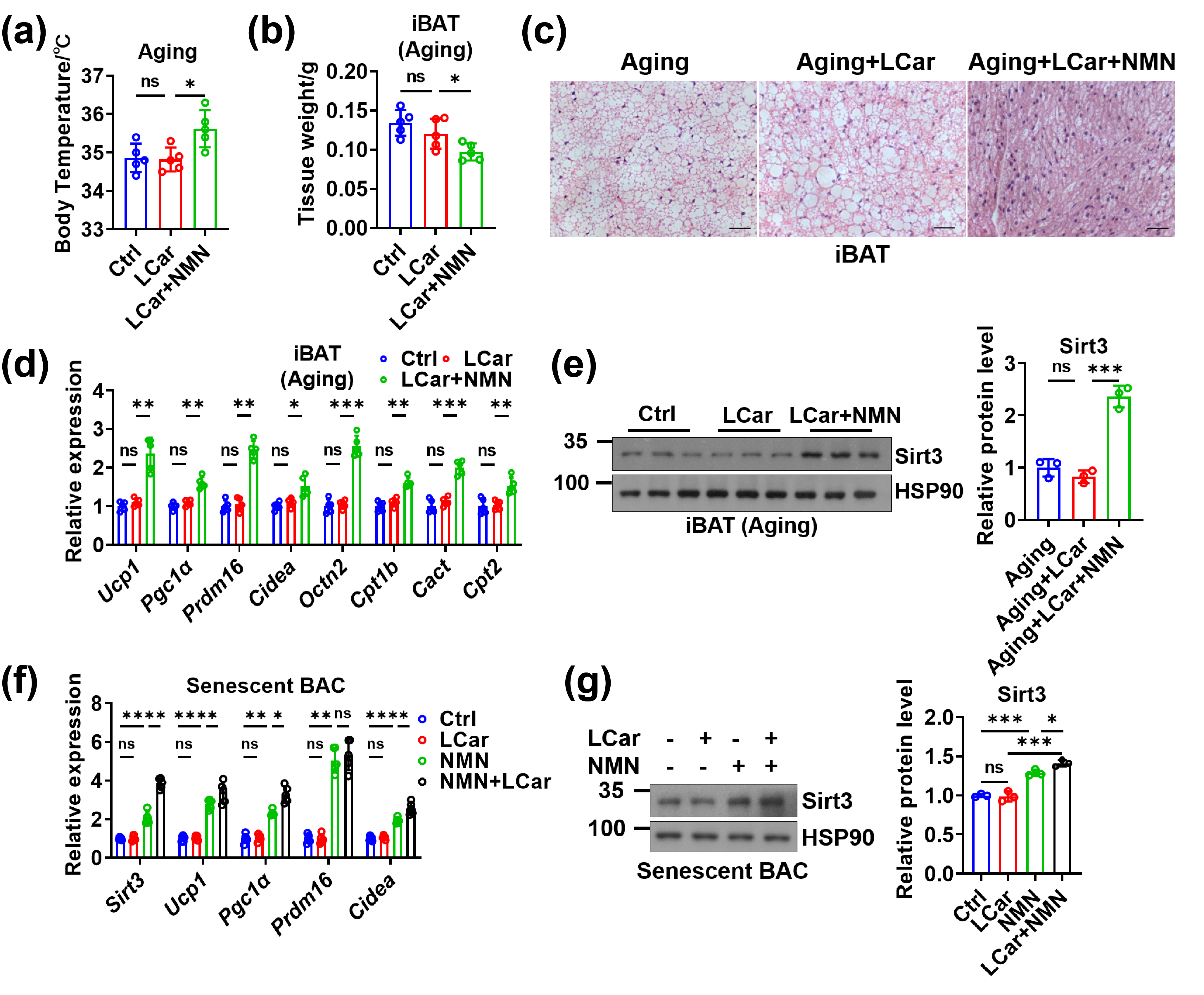


**Figure S6.** Combination of NMN and LCar improves thermogenesis and ACar metabolism of senescent brown adipocytes. (a) Core body temperature of aging mice, aging mice treated with LCar, and aging mice treated with LCar and NMN after cold exposure for 48 h (n=5). (b) iBAT weight of aging mice, aging mice treated with LCar, and aging mice treated with LCar and NMN after cold exposure (n=5). (c) Representative H&E staining of iBAT from aging mice, aging mice treated with LCar, and aging mice treated with LCar and NMN after cold exposure. (d) Relative mRNA levels of *Ucp1*, *Pgc1α*, *Prdm16*, *Cidea*, *Octn2*, *Cpt1b*, *Cact*, and *Cpt2* in iBAT of aging mice, aging mice treated with LCar, and aging mice treated with LCar and NMN after cold exposure (n=5). (e) Western blots of Sirt3 and HSP90 and relative protein level of Sirt3 in iBAT of aging mice, aging mice treated with LCar, and aging mice treated with LCar and NMN after cold exposure (n=3). (f) Relative mRNA levels of *Sirt3*, *Ucp1*, *Pgc1α*, *Prdm16*, and *Cidea* in senescent BACs treated with LCar, NMN, and combination of NMN and LCar (n=5). (g) Western blots of Sirt3 and HSP90 and relative protein level of Sirt3 in senescent BACs treated with LCar, NMN, and combination of NMN and LCar (n=3). Data are presented as the mean ± SEM and n indicates the number of biologically independent experiments. *p < 0.05; **p < 0.01; ***p < 0.001; ns, not statistically significant (one-way ANOVA).

**Table S1 Primer sequences used for qRT-PCR.**

| Primer | Primer sequences (5'to3') |
| --- | --- |
| *Sirt3-F* | ATCCCGGACTTCAGATCCCC |
| *Sirt3-R* | CAACATGAAAAAGGGCTTGGG |
| *Ucp1-F* | AGGCTTCCAGTACCATTAGGT |
| *Ucp1-R* | CTGAGTGAGGCAAAGCTGATTT |
| *Prdm16-F* | CCACCAGCGAGGACTTCAC |
| *Prdm16-R* | GGAGGACTCTCGTAGCTCGAA |
| *Cidea-F* | TGACATTCATGGGATTGCAGAC |
| *Cidea-R* | GGCCAGTTGTGATGACTAAGAC |
| *Pgc1α-F* | TATGGAGTGACATAGAGTGTGCT |
| *Pgc1α-R* | CCACTTCAATCCACCCAGAAAG |
| *Octn2-F* | AAGACCTGCAGGAAGCTGAA |
| *Octn2-R* | TCCTTGTTTTTCGTGGGTGT |
| *Cact-F* | GGTGGCTGTCCAGACAAACT |
| *Cact-R* | TCCGTTTAAGAACCTCCTGG |
| *CPT2-F* | TCTTCCTGAACTGGCTGTCA |
| *Cpt2-R* | GTACCCACCATGCACTACCA |
| *Cpt1b-F* | GCTGCTTGCACATTTGTGTT |
| *Cpt1b-R* | TGAGTGACTGGTGGGAAGAA |
| *Pparα-F* | TGCAAACTTGGACTTGAACG |
| *Pparα-R* | GATCAGCATCCCGTCTTTGT |
| *Vegf-F* | AGGGCAGAATCATCACGAAGT |
| *Vegf-R* | AGGGTCTCGATTGGATGGCA |
| *Bnip3-F* | CAGAGCGGGGAGGAGAAC |
| *Bnip3-R* | GAGGCTGGAACGCTGCTC |
| *β-actin-F* | AACCGTGAAAAGATGACCCAGAT |
| *β-actin-R* | CACAGCCTGGATGGCTACGTA |
